# Combined inactivation of the *Clostridium cellulolyticum *lactate and malate dehydrogenase genes substantially increases ethanol yield from cellulose and switchgrass fermentations

**DOI:** 10.1186/1754-6834-5-2

**Published:** 2012-01-04

**Authors:** Yongchao Li, Timothy J Tschaplinski, Nancy L Engle, Choo Y Hamilton, Miguel Rodriguez, James C Liao, Christopher W Schadt, Adam M Guss, Yunfeng Yang, David E Graham

**Affiliations:** 1 Biosciences Division, Oak Ridge National Laboratory, PO Box 2008, MS-6038, Oak Ridge, TN 37831-6038, USA; 2 BioEnergy Science Center, Oak Ridge National Laboratory, Oak Ridge, TN 37831, USA; 3 Department of Chemical and Biomolecular Engineering, University of California, Los Angeles, Los Angeles, CA 90095, USA; 4 Department of Microbiology, University of Tennessee, Knoxville, TN 37996-0845, USA

**Keywords:** Cellulose, ethanol, biofuel, *Clostridium cellulolyticum*, metabolic engineering, fermentation

## Abstract

**Background:**

The model bacterium *Clostridium cellulolyticum *efficiently degrades crystalline cellulose and hemicellulose, using cellulosomes to degrade lignocellulosic biomass. Although it imports and ferments both pentose and hexose sugars to produce a mixture of ethanol, acetate, lactate, H_2 _and CO_2_, the proportion of ethanol is low, which impedes its use in consolidated bioprocessing for biofuels production. Therefore genetic engineering will likely be required to improve the ethanol yield. Plasmid transformation, random mutagenesis and heterologous expression systems have previously been developed for *C. cellulolyticum*, but targeted mutagenesis has not been reported for this organism, hindering genetic engineering.

**Results:**

The first targeted gene inactivation system was developed for *C. cellulolyticum*, based on a mobile group II intron originating from the *Lactococcus lactis *L1.LtrB intron. This markerless mutagenesis system was used to disrupt both the paralogous L-lactate dehydrogenase (*Ccel_2485; ldh*) and L-malate dehydrogenase (*Ccel_0137; mdh*) genes, distinguishing the overlapping substrate specificities of these enzymes. Both mutations were then combined in a single strain, resulting in a substantial shift in fermentation toward ethanol production. This double mutant produced 8.5-times more ethanol than wild-type cells growing on crystalline cellulose. Ethanol constituted 93% of the major fermentation products, corresponding to a molar ratio of ethanol to organic acids of 15, versus 0.18 in wild-type cells. During growth on acid-pretreated switchgrass, the double mutant also produced four times as much ethanol as wild-type cells. Detailed metabolomic analyses identified increased flux through the oxidative branch of the mutant's tricarboxylic acid pathway.

**Conclusions:**

The efficient intron-based gene inactivation system produced the first non-random, targeted mutations in *C. cellulolyticum*. As a key component of the genetic toolbox for this bacterium, markerless targeted mutagenesis enables functional genomic research in *C*. *cellulolyticum *and rapid genetic engineering to significantly alter the mixture of fermentation products. The initial application of this system successfully engineered a strain with high ethanol productivity from cellobiose, cellulose and switchgrass.

## Background

Cellulose is the most abundant renewable feedstock on earth for biofuel production [1]. However, the economic feasibility and sustainability of cellulosic biofuels are limited by the inefficient breakdown of recalcitrant cellulose fibers into sugars [2] and their fermentation into biofuels and other metabolites [3]. While this biological conversion can be achieved in separate steps, a more economical solution will be to combine the processes into a single step, termed consolidated bioprocessing (CBP) [4]. An efficient CBP scheme could either exploit a consortium of cellulolytic and ethanologenic microorganisms, or it could use a single microorganism with both activities. No natural microorganisms have been identified that possess all the necessary characteristics of an ideal CBP strain for industrial applications [5], therefore genetic engineering will likely be required for constructing an efficient CBP microorganism.

*Clostridium cellulolyticum *is a model mesophilic clostridial species for studying cellulose and hemicellulose degradation, and an excellent candidate for CBP strain development based on its ability to ferment its hydrolysis products to ethanol and organic acids [6,7]. *C. cellulolyticum *has previously been transformed by electroporation [8], enabling random transposon-based mutagenesis [9], and laying the groundwork for more advanced genetic manipulation. Broadly, our goal is to build a genetic platform for the functional genomic analysis and genetic engineering of *C. cellulolyticum *to better understand the genetic and metabolic processes that would be required to enhance biofuel production.

Rational metabolic engineering includes three primary strategies to divert carbon and electron flow from byproducts to increase ethanol yield and purity: introduce heterologous genes, increase the expression of native genes, and disrupt genes required for competing pathways in mixed fermentations [10]. Examples of the first strategy include the heterologous expression of the *Zymomonas mobilis *pyruvate decarboxylase and alcohol dehydrogenase genes to increase ethanol production in monosaccharide-fermenting *Escherichia coli *[11] and later *C. cellulolyticum *[12]. The heterologous expression of five genes in *C. cellulolyticum *converted pyruvate to isobutanol [13]. Targeted mutagenesis experiments have also led to significant strain improvements. Deletions of the L-lactate dehydrogenase (*ldh*), phosphotransacetylase (*pta*) and acetate kinase (*ack*) genes of *Thermoanaerobacterium saccharolyticum *substantially increased the yield of ethanol from glucose and xylose fermentations, creating a homoethanologenic strain [5]. However, a deletion of the *pta *gene of *Clostridium thermocellum *eliminated acetate production but had minimal effect on ethanol production from cellulose fermentation [14].

Targeted gene inactivation has never been reported for *C. cellulolyticum*, which significantly impedes genetic engineering efforts. Here, we describe a targeted gene knockout system for *C. cellulolyticum *based on a mobile group II intron originating from the *Lactococcus lactis *L1.LtrB intron, which has been successfully used in several clostridial species to disrupt genes [15-17]. This intron is a fragment of catalytic RNA that inserts into double-stranded DNA in a site-specific manner called retrohoming [18]. The intron used in this study consists of a 915-bp *L. lactis *L1.LtrB-ΔORF intron flanked by short 5' and 3' exons and a downstream *ltrA *gene encoding a protein with endonuclease and reverse transcriptase activities [19]. The target specificity is primarily determined by base pairing of intron RNA and target site DNA, and intron insertion does not require host-supplied factors; therefore, the intron can be easily modified to insert into virtually any DNA sequence in principle [20]. However, a replicative plasmid and a strong promoter are required to drive the transcription of the intron and *ltrA *genes in each host strain [21].

As an initial step to increase ethanol production by *C. cellulolyticum *during cellulosic fermentation, we decided to disrupt the production of lactate, one of the cells' major fermentation products, by inactivating the lactate dehydrogenase (*ldh*) gene. *C. cellulolyticum *has two paralogs of the *ldh *gene that were both originally predicted to encode L-lactate dehydrogenase (LDH) enzymes: *Ccel_0137 *and *Ccel_2485*. An intron-based gene inactivation system was used to create markerless mutations in both genes to clarify their metabolic roles during growth on complex carbohydrates. The method applied here did not require the use of a retrotransposition-activated marker for integrant selection [22], allowing the rapid introduction of both *Ccel_0137 *and *Ccel_2485 *mutations in a single *C. cellulolyticum *strain.

## Results and Discussion

### Construction of *Ccel_0137 *and *Ccel_2485 *mutations in *C. cellulolyticum*

**Figure 1:**
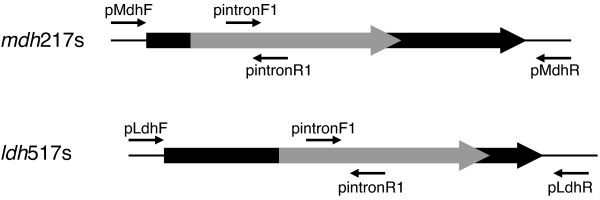
**Diagram of the *mdh *and *ldh *genes with markerless intron insertions in the sense orientation**. The 933-bp *mdh *gene (*Ccel_0137*) contains an intron inserted between bases 216 and 217. The 951-bp *ldh *gene (*Ccel*_2485) contains an intron inserted between bases 516 and 517.

**Figure 2:**
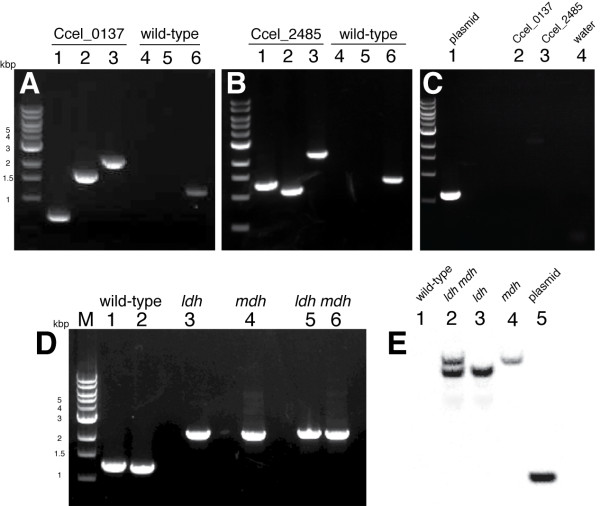
**PCR and Southern blot experiments showed that group II intron-based vectors efficiently targeted the *Clostridium cellulolyticum mdh *and *ldh *genes in pure cultures**. **(A) **Primers MdhF/intronR1 (5' junction) and intronF1/MdhR (3' junction) produced bands from the *Ccel_0137 *mutant cells (lanes 1 and 2) but not from wild-type (lanes 4 and 5). Primers MdhF/MdhR amplified a single band from the mutant (lane 3) that is 915 bp larger than the wild-type (lane 6). **(B) **Primers LdhF/intronR1 (5' junction) and intronF1/LdhR (3' junction) produced bands in the *Ccel_2485 *mutant cells (lanes 1 and 2) but not in the wild-type (lanes 4 and 5). Primers LdhF/LdhR, amplified a single band from the mutant (lane 3), which is 915 bp larger than the wild-type (lane 6). **(C) **Amplifications using plasmid-specific primers pWH199F2 and pintronR1 confirmed plasmid curing. Lane 1, positive control (plasmid); lane 2, *Ccel_0137 *mutant; lane 3, *Ccel_2485 *mutant; lane 4, negative control. **(D) **Amplification from wild-type DNA using primers LdhF-R (lane 1) and MdhF-R (lane 2) produced low molecular weight products. Genes containing insertions were amplified from the *ldh *mutant using primers LdhF-R (lane 3) and from the *mdh *mutant using primers MdhF-R (lane 4), producing larger products. The same size PCR products were obtained in amplifications from *ldh mdh *mutant DNA using primers LdhF-R (lane 5) and MdhF-R (lane 6). **(E) **A Southern blot using an intron-specific probe confirmed the intron insertions in DNA digested with *Eco*RI. No band was detected in the chromosomal DNA of wild-type cells (lane 1), while two bands in the *ldh mdh *mutant (lane 2) correspond to bands in the *ldh *mutant (lane 3) and the *mdh *mutant (lane 4). No band corresponding to the plasmid (lane 5) was identified in any of the plasmid-cured strains.

Insertion mutations in the paralogous *ldh *genes, *Ccel_0137 *and *Ccel_2485*, were constructed using pLyc1217Er-based vectors that encoded targeted introns (targetrons) specific for each gene. *C. cellulolyticum *cells transformed with these vectors produced erythromycin-resistant colonies on agar plates. Colony PCR was performed using forward and reverse primers flanking the intron insertion sites of the targeted genes to screen transformants for the desired gene insertions (Figure 1). The proportion of transformants containing markerless chromosomal insertion mutations depends on the retargeting efficiency [16]. Eight colonies transformed with pLyc1217Er0137 were screened by PCR for insertion in the *Ccel_0137 *gene: seven were wild-type, whereas one contained both an intron insertion allele (2,164-bp PCR product) and the wild-type allele (1,249-bp PCR product) (Additional file 1). This colony was streaked on an agar plate containing 15 μg/ml erythromycin, and colony PCR was performed until a single intron-inserted PCR band was identified in the tested colonies, demonstrating the homogeneity of the purified *Ccel_0137 *mutant. Similarly, the second *ldh *paralog was disrupted using vector pLyc1217Er2485, and eight colonies were screened for insertion in the *Ccel_2485 *gene: four showed an intron insertion allele (2,197-bp PCR product) and the wild-type allele (1,282-bp PCR product), whereas two contained a single intron insertion allele (2,197-bp PCR product) (Additional file 1). Colony 4 became the *Ccel_2485 *mutant used for further analysis. Colony PCR was performed to confirm the correct insertion sites and intron orientation of the isolated mutant strains (Figure 2A,B). PCR products from the mutants amplified by primers MdhF/MdhR and LdhF/LdhR were sequenced, verifying the correct intron insertions in the mutant strains (Additional file 2).

Because an intron inserted in the sense orientation could be spliced from the precursor mRNA by the plasmid-encoded protein LtrA [19] resulting in a wild-type mRNA, the plasmid was cured from all of the mutants to achieve an unconditional knockout before the fermentation and analytical experiments. The knockout plasmids pLyc1217Er0137 and pLyc1217Er2485 were cured from *Ccel_0137 *and *Ccel_2485 *mutants by serial passage in non-selective medium without erythromycin (Figure 2C). These plasmid-cured mutants were unable to grow in liquid medium supplemented with erythromycin, demonstrating efficient plasmid curing and markerless mutagenesis. The plasmid-cured *Ccel_2485 *mutant was transformed with plasmid pLyc1217Er0137, and a strain carrying an intron insertion in the *Ccel_0137 *gene was isolated (Figure 2D). Therefore, multiple gene inactivations in the same *C. cellulolyticum *strain could be introduced sequentially by pLyc1217Er-based plasmids targeting different genes.

Southern blots were performed on genomic DNA from the *Ccel_0137*, *Ccel_2485 *and double mutants after plasmid curing, using an intron-specific probe (Figure 2E). Both single mutants contained a single intron insertion in the chromosome with expected sizes of 4.1 or 4.9 kbp. The double mutant contained both bands. The knockout vector pLyc1217Er showed a band of 1.5 kbp, which was not present in the mutants. No band was detected in chromosomal DNA from wild-type cells. These hybridization experiments confirmed specific gene disruption, with no residual vector remaining in the mutants.

These *Ccel_0137 *and *Ccel_2485 *mutants are the first strains of *C. cellulolyticum *produced by targeted mutagenesis. The strong *Clostridium pasteurianum *ferredoxin promoter enabled intron and *ltrA *transcription in *C. cellulolyticum*. This mode of accurate and specific chromosomal insertions had a sufficiently high efficiency that mutants could be readily identified by screening a small number of colonies without chromosomal antibiotic counter selection. This high targeting efficiency is essential for *C. cellulolyticum *gene inactivation, due to the cells' low transformation efficiency. Another advantage of this intron-based knockout system is that the gene inactivation occurs in a one-step intron insertion process. The traditional gene disruption strategy based on homologous recombination usually takes two steps: one single crossover followed by a second crossover. That strategy requires at least two selectable markers, and a large number of colonies often need to be screened to identify a mutant [23]. The intron-based gene knockout system used to construct these mutations was especially advantageous for the manipulation of these obligately anaerobic clostridia. The lack of a selectable marker in the intron and the ease of plasmid curing will also accelerate future efforts to produce strains with multiple mutations, introduced sequentially using pLyc1217Er. Because several nearly identical intron fragments would be present on the genome after multiple intron insertions, one possible problem is that homologous recombination might occur among these introns, causing genetic instability. Therefore, the long-term goal of genetic engineering of *C. cellulolyticum *using a multiple-intron insertion strategy may require the inactivation of the organism's *recA *gene.

### Characterization of *Ccel_0137 *and *Ccel_2485 *single mutants

**Figure 3:**
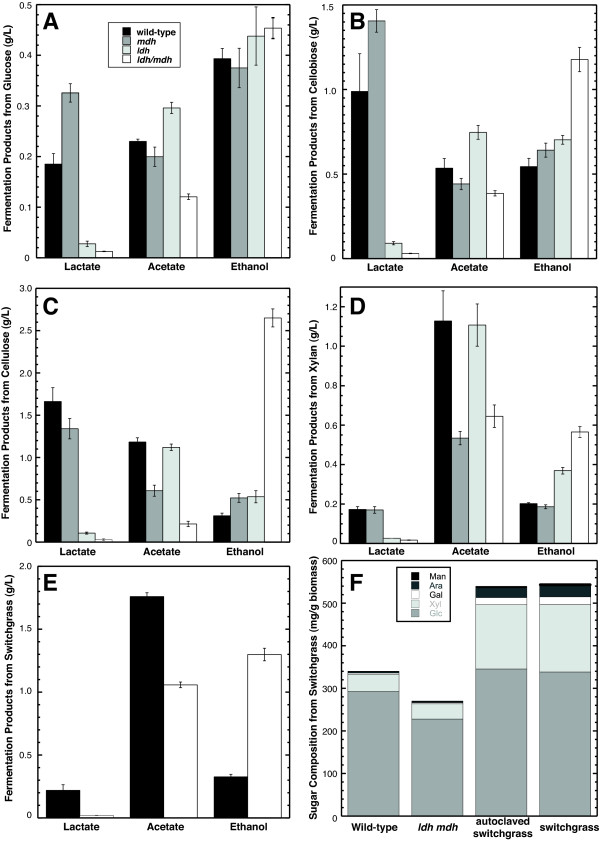
**Fermentation end-product profiles of *Clostridium cellulolyticum *strains grown on various carbon sources**. The *C. cellulolyticum *mutants were grown on defined VM media with D-glucose (5 g/l) in **(A)**, D-cellobiose (5 g/l) in **(B) **crystalline Avicel cellulose (10 g/l) in **(C)**, birch wood xylan (5 g/l) in **(D)**, and acid-pretreated switchgrass (10 g/l) in **(E)**. Lactate, acetate and ethanol concentrations were measured by high-performance liquid chromatography (HPLC), and the error bars represent standard deviations of measurements from three replicate cultures (except two replicate cultures were grown on xylan). Lactate concentrations were significantly lower in *ldh *and *ldh mdh *mutants compared to wild-type for growth on each substrate (*P *< 0.05, determined by analysis of variance (ANOVA) for (A-C) and by Student's t test for (D)). Acetate concentrations were also significantly lower in *mdh *and *ldh mdh *mutants compared to wild-type (*P *< 0.05). Ethanol concentrations were significantly higher in the *ldh mdh *mutant compared to wild-type (*P *< 0.05) for each substrate except glucose (A). **(F) **Shows results from quantitative saccharification of the residual biomass from wild-type and *ldh mdh *mutant cultures grown on acid-pretreated switchgrass and the acid-pretreated switchgrass substrate (before and after autoclaving).

**Figure 4:**
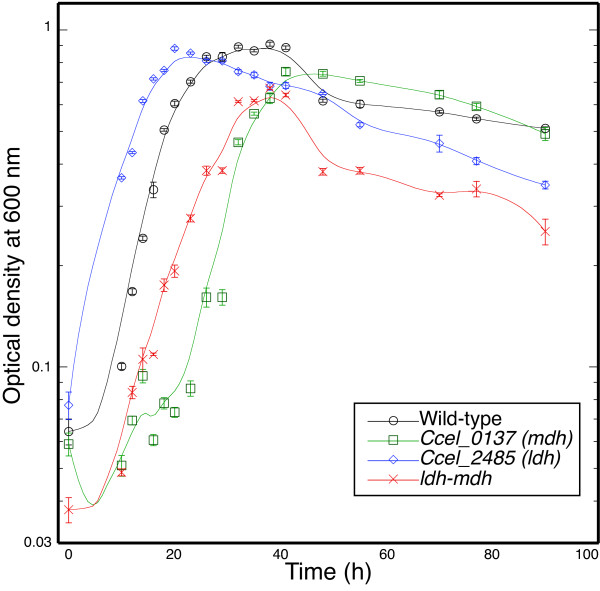
**Growth curves for wild-type and mutant *Clostridium cellulolyticum *strains grown in defined VM medium with cellobiose (5 g/l)**. The specific growth rates for cells in exponential growth phases were 0.20 ± 0.006/h (wild-type), 0.19 ± 0.004/h (*Ccel_0137 *(*mdh*) mutant), 0.15 ± 0.006/h (*Ccel_2485 *(*ldh*) mutant), and 0.11 ± 0.002/h (*ldh mdh *mutant). The mean and standard deviation are shown for three cultures at each time point.

High-performance liquid chromatography (HPLC) analysis of fermentation products from *Ccel_2485 *mutant cells grown in defined VM medium with cellobiose showed that the lactate concentration decreased significantly to 0.09 ± 0.01 g/l, less than 10% of wild-type levels (Figure 3B). A small but significant (*P <*0.0005) increase in acetate was observed for the mutant (0.75 ± 0.04 g/l versus 0.54 ± 0.06 g/l for wild-type cells). Ethanol concentrations were 30% higher in the mutant. In contrast, the *Ccel_0137 *mutant produced significantly more lactate than wild-type cells (1.4 ± 0.07 g/l versus 1.0 ± 0.22 g/l; *P *< 0.01) and 19% more ethanol than wild-type. Cultures of both mutants grew to similar maximum turbidities, with a similar exponential growth rate compared to wild-type cells (Figure 4). Only the inactivation of the *Ccel_2485 *gene produced the expected defect in lactate production, establishing that this locus encodes the primary lactate dehydrogenase.

**Figure 5:**
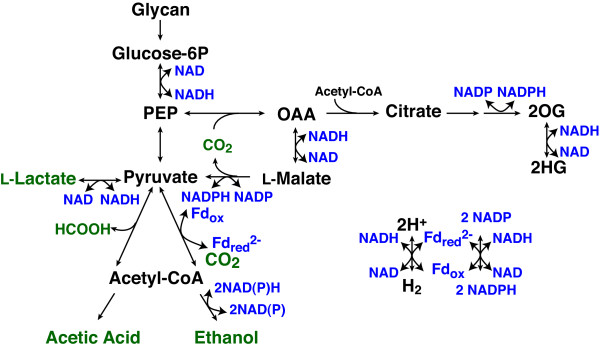
**Key metabolic pathways of glycolysis, fermentation and electron transfer to nicotinamide adenine dinucleotide phosphate (NAD(P)^+^) cofactors in *Clostridium cellulolyticum***. L-Malate dehydrogenase (MDH) catalyzes the NADH-dependent reduction of oxaloacetate (OAA), forming the L-malate intermediate, and malic enzyme catalyzes the NADP^+^-dependent oxidation and decarboxylation of the intermediate to produce pyruvate and NADPH. In a second transhydrogenase system, isocitrate dehydrogenase catalyzes 2-oxoglutarate (2OG) and NADPH formation, while a putative 2-oxoacid dehydrogenase could catalyze the NADH-dependent formation of 2-hydroxyglutarate (2HG). Ferredoxin (Fd) reduced by the pyruvate:ferredoxin oxidoreductase enzyme can be oxidized to produce H_2 _or coupled to the NADH-dependent reduction of NADP^+ ^catalyzed by the iron-sulfur flavoprotein complex NfnAB. Fermentation products are shown in green, and electron transfer cofactors are shown in blue.

The *Ccel_2485 *gene is an ortholog of the *Clostridium thermocellum ldh *(*Cthe_1053*) gene that was characterized previously (Additional file 3) [24]. The paralogous *Ccel_0137 *gene shares the same orientation as the adjacent *Ccel_0138 *gene on the *C. cellulolyticum *chromosome, forming a putative operon. The latter locus is predicted to encode a nicotinamide adenine dinucleotide phosphate (NAD(P))^+^-dependent malic enzyme, which catalyzes the oxidative decarboxylation of L-malate producing NAD(P)H and pyruvate. The cofactor binding site of the *Ccel_0138 *protein more closely resembles the sequence of NADP^+^-dependent malic enzymes than NAD^+^-dependent proteins [25], and the *Ccel_0138 *protein is probably an ortholog of the NADP^+^-dependent malic enzyme that was characterized previously from *C. thermocellum *[26]. Both the sequence and organization of the *Ccel_0137 *and *Ccel_0138 *genes are conserved in *C. thermocellum *(*Cthe_0345 *and *Cthe_0344*). Therefore the *Ccel_0137 *protein was likely to function as a L-malate dehydrogenase (MDH). The MDH enzyme was proposed to act in concert with the malic enzyme to convert oxaloacetate to pyruvate and transfer electrons from NADH to form NADPH (Figure 5).

Malate dehydrogenase and malic enzyme activities were measured in cell-free extracts from wild-type, *Ccel*_*2485 *and *Ccel*_*0137 *mutants (Table 1). The *Ccel_0137 *mutant demonstrated 28% of the NADH-dependent malate dehydrogenase activity observed in wild-type cells, while activity in the *Ccel_2485 *mutant was not significantly different from wild-type. Although both mutants had significant malate dehydrogenase activity, the *Ccel_0137 *gene appears to encode the primary malate dehydrogenase. None of the extracts catalyzed the reduction of oxaloacetate using NADPH. All three strains had similar levels of malic enzyme activity (Table 1). This enzyme required NADP^+ ^and L-malate for activity: reactions containing NAD^+ ^or D-malate had 13% or 0.5% relative activity, respectively. These results indicate that the *Ccel_0137 *insertion has no polar effect on *Ccel_0138 *expression and support the model of oxaloacetate-dependent transhydrogenase activity illustrated in Figure 5. This pathway was predicted to be one of two primary modes of NADPH production for biosynthesis in *C. thermocellum *[27]. Disrupting the MDH-malic enzyme transhydrogenase system would be expected to cause an increase in flux through alternative, ferredoxin-dependent transhydrogenase systems. Both the *C. cellulolyticum *and *C. thermocellum *genomes also encode homologs of the *Clostridium kluyveri nfnAB *genes that encode a ferredoxin-dependent transhydrogenase system, which provides another source of NADPH to these organisms [28]. Ferredoxin is primarily reduced by the pyruvate:ferredoxin oxidoreductase complex. This NfnAB system catalyzes an electron bifurcation event, where the exergonic oxidation of reduced ferredoxin and NADH is coupled to the endergonic reduction of NADP^+ ^(Figure 5). Unlike *C. thermocellum*, *C. cellulolyticum *lacks homologs of the cation-translocating ferredoxin:NAD oxidoreductase (Rnf complex) [29], which conserves energy (as ion-motive force) and transfers electrons from reduced ferredoxin.

**Table 1:** Enzyme activities in cell-free extracts of *Clostridium cellulolyticum *wild-type and mutant cultures

Strain	Specific activity (U/mg)		
	
	MDH	ME	LDH
Wild-type	1.66 ± 0.088	0.30 ± 0.020	< 0.007
*Ccel_0137*::LtrB (*mdh*)	0.47 ± 0.056**	0.25 ± 0.013*	ND
*Ccel_2485*::LtrB (*ldh*)	1.54 ± 0.36	0.29 ± 0.019	NA
(*ldh mdh*)	NA**	0.44 ± 0.019**	< 0.055

Mean and standard deviations of enzymatic specific activities are shown from at least three replicate assays. Continuous spectrophotometric assays were performed aerobically at ambient temperature (22°C), using 10 to 20 μg of protein sample in 1-ml reaction mixtures; 1 U of activity catalyzed the oxidation of 1 μmol of NAD(P)H per minute (MDH and LDH), or the reduction of 1 μmol of NADP^+ ^per minute (ME). Specific activities were corrected for substrate-independent NAD(P)H oxidation. Malate dehydrogenase (MDH, EC 1.1.1.37) and malic enzyme (ME, EC 1.1.1.40) activities were measured as described in the Methods section, using NADH and NADP^+ ^cofactors, respectively. No MDH activity was detected in the *ldh mdh *extracts (NA). Lactate dehydrogenase (LDH, EC 1.1.1.27) activity was determined as for MDH activity, using 1 mM pyruvate, 1 mM fructose-1,6-diphosphate and 0.2 mM NADH. No pyruvate-dependent NADH oxidation was detected in any sample: limits of detection are shown for wild-type and *ldh mdh *extracts.

Values significantly different from wild-type activity (*P < 0.05; **P < 0.005) were calculated using analysis of variance (ANOVA).

ND = not determined.

The family of 2-hydroxyacid dehydrogenases includes both lactate and malate dehydrogenases, and a single discriminating amino acid substitution (Gln102Arg) converted the *Geobacillus stearothermophilus *lactate dehydrogenase into an efficient malate dehydrogenase [30]. The corresponding amino acids are Gln86 in the Ccel_2485 protein and Arg81 in the Ccel_0137 protein. Only five amino substitutions in the *E. coli *MDH, including the Arg to Gln substitution, increased the enzyme's specificity for pyruvate by a factor of 4.6 × 10^9 ^[31]. Together with the biochemical data described above, these comparisons suggest that both enzymes catalyze lactate and malate dehydrogenase reactions, although the *Ccel*_*2485 *gene encodes the primary lactate dehydrogenase and the *Ccel_0137 *gene encodes the primary malate dehydrogenase enzyme. The clostridial LDH and MDH proteins share significant sequence similarity and have overlapping substrate specificity, therefore they could have diverged following the duplication of a single ancestral gene in the clostridial lineage. However, a phylogeny that includes more homologs does not support this hypothesis (Additional file 3). Instead, most LDH and MDH proteins appear to derive from separate linages, although bootstrap support is weak for deeply branching nodes in this tree. LDH activity levels were below the limits of detection for all *C. cellulolyticum *cell-free extracts (Table 1), potentially due to low levels of the enzyme at the tested growth stage or a requirement for an unrecognized allosteric activator. Future studies using purified enzymes will be required to measure specificity constants and elucidate the structural basis for substrate discrimination by these two enzymes.

### Characterization of a *Ccel_2485 (ldh) *and *Ccel_0137 (mdh) *double mutant

Residual MDH and LDH activities in the single mutants suggested the two proteins have partially overlapping substrate specificities, which prevented the complete redirection of carbon flow in these strains. The markerless mutagenesis strategy developed here enabled the rapid construction of an *ldh mdh *double mutant. This mutant produced negligible lactate during fermentation (Figure 3), and had no detectable MDH activity although malic enzyme activity was significantly higher (Table 1). Most importantly, ethanol production doubled during growth on minimal VM medium with cellobiose (Figure 3). These results confirm that the paralogs are responsible for almost all lactate production in *C. cellulolyticum*.

### Effects of *mdh *and *ldh *mutations on fermentation product mixtures from complex substrates

*C. cellulolyticum *wild-type and mutant strains were grown in defined VM and MTC media with different carbon sources to compare the metabolic products (Figure 3 and Additional file 4). Wild-type *C. cellulolyticum *grew poorly in defined glucose medium, fermenting only 26% of the sugar. Cultures of the *ldh mdh *double mutant fermented 22% of the glucose and produced only 7% of the lactate secreted by wild-type cells. This lactate concentration was 54% lower than values measured from *ldh *mutant cultures, which fermented 27% of the glucose. The residual lactate in the *ldh mdh *mutant could be D-lactate, formed by the putative D-2-hydroxyacid dehydrogenase Ccel_3425 [32]. Acetate concentrations in the *ldh mdh *mutant cultures were 50% of wild-type levels, while ethanol production was slightly higher.

In contrast, all four strains grew vigorously in defined medium with cellobiose. Wild-type, *mdh *and *ldh *mutant cells grew with doubling times of approximately 3.5 h, while the *ldh mdh *mutant grew with a doubling time of 6.3 h and reached a lower maximum turbidity (Figure 4). Wild-type cells fermented 54% of the cellobiose to produce primary fermentation products including 12 mM ethanol. The *ldh mdh *mutant fermented the same proportion of substrate but produced significantly less acetate and more than twice the concentration of ethanol (Figure 3B). This ethanol yield is approximately 44% of the maximum theoretical conversion from 5 g/l cellobiose, and it represents 53% of the carbon and 79% of the electron equivalents in the primary fermentation products.

In addition to the primary fermentation products, *C. cellulolyticum *secretes numerous minor products that together account for approximately 3% of the carbon and electron equivalents. To identify differences in the levels of these metabolites that could indicate changes in the carbon fluxes of mutants, we used gas chromatography-mass spectrometry (GC-MS) to analyze metabolites in the cell pellets and culture supernatants of cells grown on cellobiose (Table 2). Concentrations of extracellular pyruvate were highest in the *ldh *mutant and lowest in the *mdh *mutant; however, concentrations of pyruvate-derived amino acids were similar in the supernatants of all four strains. Malate concentrations were approximately 70% lower than wild-type in both *mdh *and *ldh mdh *mutants (0.65, 0.21 and 0.18 mM, respectively). The residual malate in the double mutant could be produced by the reductive carboxylation of pyruvate (catalyzed by the malic enzyme), or by fumarase (Ccel_2421-2422), which catalyzes the addition of water to fumarate produced by the argininosuccinate lyase enzyme (Ccel_1344). The accumulation of citrate and 2-hydroxyglutarate in the *ldh mdh *mutant suggests that the lack of MDH activity causes increased flux through citrate synthase, aconitase and the NADPH-producing isocitrate dehydrogenase enzymes. The 2-oxoglutarate product could be reduced using NADH to form 2-hydroxyglutarate [33], effectively reproducing the transhydrogenase activity of malate dehydrogenase and malic enzyme (Figure 5). Isotope incorporation experiments using *Clostridium acetobutylicum *suggest flux through both the oxidative and reductive branches of the incomplete citric acid cycle that cannot be explained by canonical genes in its genome [34,35]; therefore future experiments will be required to determine the relative fluxes through both branches of the incomplete citric acid cycle in *C. cellulolyticum*.

**Table 2:** Metabolites differing in relative abundance in cell pellets and culture supernatants of *Clostridium cellulolyticum mdh*, *ldh *and *ldh mdh *mutants compared to wild-type at stationary growth phase

Compound	Cell pellets: abundance ratio to wild-type			Culture supernatants: abundance ratio to wild-type		
	
	*mdh*	*ldh*	*mdh*-*ldh*	*mdh*	*ldh*	*mdh*-*ldh*
Malic acid	0.32	1.48	0.28	0.84	1.44	0.57
2-Hydroxyglutaric acid	0.28	1.42	12.43	0.35	1.72	4.94
Valine	0.27	1.01	0.37	0.35	1.17	0.68
Glucose-1-P	0.90	1.20	0.56	0.89	1.36	0.57
Fructose-6-P	0.70	0.99	0.79	0.99	2.89	1.39
Glucose-6-P	0.39	1.11	1.12	1.41	9.21	4.69
Succinic acid	0.51	0.56	1.12	0.58	0.92	0.62
Fumaric acid	0.31	0.70	0.40	0.48	0.79	0.36
*p*-Hydroxybenzoic acid	0.53	0.65	1.24	0.46	0.98	1.77
10.13 *191 246 376 348*	0.22	0.88	0.25	0.35	1.13	0.90
Citric acid	0.12	0.96	6.03	ND	ND	ND
11.05 *191 276*	0.44	1.53	0.18	ND	ND	ND
4-Methyl-2-hydroxypentanoic acid	ND	ND	ND	0.17	0.91	2.63
Pyruvic acid	0.56	0.45	0.88	0.18	2.25	0.95
Galactose	ND	ND	ND	3.41	2.67	2.32
2,3-Butanediol	ND	ND	ND	0.29	1.76	1.92
9.97 *331 359 226*	ND	ND	ND	0.11	1.62	2.12
11.07 *191 348 320 246*	ND	ND	ND	0.73	1.46	0.44

Metabolites were analyzed by gas chromatography-mass spectrometry (GC-MS) as trimethylsilyl (TMS) derivatives, using a sorbitol internal standard [42]. Peaks that could not be assigned to a standard metabolite are identified by their chromatographic retention times (in min) and their characteristic ion peaks (*m/z *in italic text).

ND = not determined (the concentrations of these metabolites were too low to produce reliable estimates of relative abundance).

Cells grown on crystalline cellulose (10 g/l) VM medium produced similar distributions of primary fermentation products compared to cellobiose cultures (Figure 3C). Wild-type *C. cellulolyticum *fermented 36% of the cellulose, compared to 26% in the *ldh *mutant and 30% in the *mdh *mutant. The *ldh mdh *mutant fermented a remarkable 50% of the cellulose to 2.7 g/l ethanol, about nine times more ethanol than wild-type cells produced. As a result of this metabolic shift, the molar ratio of ethanol to mixed acid production increased from 0.18 in wild-type cells to 15 in the *ldh mdh *mutant. As observed for cellobiose, this ethanol includes 79% of the electron equivalents in the primary fermentation products. Similar results were obtained from growth on MTC medium containing Avicel (Additional file 4).

In medium containing 5 g/l (33 mM) D-xylose, wild-type *C. cellulolyticum *fermented 52% of the carbohydrate, primarily forming lactate and acetate (Additional file 4). Cultures of both the *ldh *and *ldh mdh *mutants produced only trace amounts of lactate, but their acetate and ethanol levels were similar to wild-type cultures. Wild-type cultures grown on 5 g/l xylan fermented 40% of the carbohydrate to form 4.4 mM ethanol, 18.8 mM acetate and 1.9 mM lactate. Cultures of both the *ldh *and *ldh mdh *mutants fermented similar proportions of the xylan, but produced significantly more ethanol (8.0 and 12.3 mM, respectively, *P *< 0.01). Both the *mdh *and *ldh mdh *mutants produced significantly less acetate (8.9 and 10.7 mM, respectively, *P *< 0.05) compared to wild-type cells. Some of this acetate may have been produced by the hydrolysis of *O*-acetyl groups from the C-2 and C-3 positions of xylose residues in hardwood xylan [36].

Switchgrass contains substantial amounts of both cellulose and hemicellulose, which includes xylose, arabinose, galactose, mannose and hexuronic acids [37]. Dilute acid pretreatment solubilizes and removes some of the hemicellulose sugars [38]. Wild-type *C. cellulolyticum *cells grown on pretreated switchgrass consumed 37% of the switchgrass sugars, compared to 50% hydrolyzed by the *ldh mdh *mutant, measured by quantitative saccharification of the residual substrate (Figure 3F). Both strains preferentially hydrolyzed the monosaccharides from hemicellulose compared to cellulose. The product mixtures from the two cultures resembled those from xylan medium (Figure 3D, E): the wild-type cells produced significantly more acetate than the mutant (29 versus 18 mM, *P *< 0.0001) and the mutant produced more ethanol (28 versus 7.1 mM, *P <*0.0005). The fourfold increase in ethanol production by the double mutant reflects a substantial diversion of both carbon and electrons from acetate and lactate to ethanol.

### Construction of conditional mutations in *Ccel_2137 *(*pta*) and *Ccel_2136 *(*ack*)

Disruption of *ldh *and *mdh *eliminated lactate as a primary fermentation product and caused an unexpected decrease in acetate production. Nevertheless, acetate was still a significant fermentation product. To further shift fermentation towards ethanol, genes involved in acetate production were targeted for inactivation in the wild-type strain. However, using multiple approaches, no mutants were isolated with unconditional disruptions in either *Ccel_2137 *(*pta*) or *Ccel_2136 *(*ack*) genes. Retrotransposon vectors were constructed targeting *pta *and *ack *in the sense orientation and transformed in *C. cellulolyticum *(Additional file 2). From these transformations, strains were isolated that contain retrotransposon insertions into *pta *and *ack *(Additional file 5) in the presence of the plasmid-encoded LtrA, which catalyzes mRNA splicing [19] and enables translation to produce functional Pta and Ack proteins. These strains fermented cellobiose, primarily producing lactate and 30% less acetate than wild-type cells; however, they were unable to grow on VM medium with cellulose as the sole carbon source. Despite repeated attempts, the plasmid could not be cured from these mutants to eliminate LtrA production and mRNA splicing, suggesting that *pta *and *ack *may be essential under the conditions tested. The mRNA can only be spliced when the intron inserts in the sense orientation, so three additional plasmids were constructed to disrupt *pta *in the antisense orientation (Additional file 2). When these plasmids were transformed into *C. cellulolyticum*, hundreds of antibiotic-resistant colonies were screened and retrotransposition occurred, but no isolate showed PCR products consistent with a viable haploid or homozygous *pta *mutant (Additional file 5), further suggesting that acetate production is essential under the conditions tested [39]. One possible explanation for the apparent essentiality of *pta *and *ack *in *C. cellulolyticum *is that H_2 _production could deplete the pool of reduced ferredoxin and NAD(P)H cofactors, forcing the cells to make fermentation products more oxidized than ethanol. Future experiments could determine the effects of the conditional *pta *and *ack *mutations on H_2 _production and electron-transfer cofactor pools, in order to infer why unconditional mutants could not be isolated using the present growth conditions.

## Conclusions

The intron-based targeted mutagenesis method proved to be an efficient genetic tool to interrogate fermentative pathways in *C. cellulolyticum*. The markerless retrotransposition strategy enabled the rapid disruption of *Ccel_2485 *and *Ccel_0137 *genes in *C. cellulolyticum*, and their combination resulted in a strain that produced remarkably high yields of ethanol during polysaccharide and switchgrass fermentations. These experiments distinguished the primary LDH and MDH paralogs, while illustrating their partially overlapping substrate specificity *in vivo*. The synergy between *ldh *and *mdh *mutations all but eliminated lactate production, as expected, and significantly decreased acetate production. These cells produced substantially more ethanol than *C. cellulolyticum *strains that expressed the heterologous *Z. mobilis *pyruvate decarboxylase and alcohol dehydrogenase proteins [12]. The *C. cellulolyticum *strains also secreted a number of minor fermentation products, identified by metabolomic analyses that improve our understanding of carbon flux and applicable pathways, while providing constraints for future metabolic models. Establishing complete carbon and electron balances will be essential to modeling and manipulating CBP fermentations using these organisms.

## Methods

### Medium and culture conditions

*E. coli *TOP10 cells (Invitrogen, Grand Island, NY) were used for cloning and were grown at 37°C in LB medium supplemented with 50 μg/ml kanamycin or 15 μg/ml chloramphenicol as appropriate. *C. cellulolyticum *H10 was cultured at 34°C anaerobically in modified VM medium [13] or MTC medium [40], with various carbon sources. For complex medium, the modified VM medium was supplemented with 2.0 g/l yeast extract. For agar plates, 1.0% (weight/volume) of Bacto agar (BD, Franklin Lakes, NJ) was added to the medium. The modified VM medium was prepared anaerobically and was supplemented with 15 μg/ml erythromycin as appropriate. A list of all *C. cellulolyticum *strains is presented in Additional file 6.

### Plasmid **construction**

The *E. coli*-*C. cellulolyticum *shuttle vector used in this study was pWH199, which was modified from plasmid pAT187 as described previously [13]. To insert the intron into pWH199 downstream of a *Clostridium pasteurianum *ferredoxin (Fd) promoter [41], a DNA fragment containing the intron and *ltrA *was amplified from pJIR750ai (Sigma-Aldrich, St. Louis, MO) by PCR using primers pJIR750aiXmaIF and pJIR750aiXhoIR (Additional file 7). The PCR product was digested with *Xma*I and *Xho*I enzymes and then ligated into pWH199 treated with the same enzymes, resulting in plasmid pLyc1217Er. The correct construction of pLyc1217Er was verified by sequencing. The intron integration sites were chosen by calculating all possible sites for insertions into *Ccel_0137 *and *Ccel_2485*, using an online intron design tool at http://www.clostron.com[16]. The program predicted multiple intron insertion sites across the genes. Based on the consideration of both optimal gene inactivation and efficient insertion, a sense integration site 216 bp downstream of the start codon was chosen for *Ccel_0137*, and a sense integration site 516 bp downstream of the start codon was chosen for *Ccel_2485*. Four PCR primers for each integration design, IBS, EBS1d, EBS2 and EBSu were created by the online intron design tool. To insert the intron to the targeted genes, a 340-bp *Xma*I-*Bsr*GI intron fragment was amplified by a one-step crossover PCR, using external primers IBS and EBS1d and internal primers EBS2 and EBSu. The PCR template was a 613-bp DNA fragment amplified with primers pWH199F2 and pintronR1 using pLyc1217Er as the template. The 340-bp intron fragments were ligated into pLyc1217Er treated by *Xma*I and *Bsr*GI to form pLyc1217Er0137 and pLyc1217Er2485. All correct constructs targeting different genes were verified by sequencing using pintronR1 as the primer. Insertion vectors targeting the *Ccel_2137 *(*pta*) and *Ccel_2136 *(*ack*) genes were similarly constructed; these are described in Additional file 2.

### Transformation

The plasmids were transformed into wild-type *C. cellulolyticum *by electroporation as previously described [8] with modifications. Cells were grown in complex modified VM medium containing 5 g/l cellobiose and 2 g/l yeast extract for 17 to 24 hours to reach early to middle log phase (optical density at 600 nm of 0.3 to 0.5). The transformation was performed at 4°C under anaerobic conditions. The cells were harvested and washed twice with ice-cold anoxic electroporation buffer containing 270 mM sucrose, 1 mM MgCl_2 _and 5 mM sodium phosphate buffer, pH 7.4. The washed cells were resuspended in electroporation buffer and stored on ice. The plasmid DNA was treated with MspI methyltransferase (New England Biolabs, Ipswich, MA) for 3 h, followed by purification with the DNA Clean and Concentrator Kit (Zymo Research, Irvine, CA). For each transformation, a 50-μl cell suspension was mixed with 2.0 μg of methylated plasmid DNA. The cells were electroporated in 2-mm gap electroporation cuvettes (BTX, Holliston, MA) with a MicroPulser electroporator (Bio-Rad, Hercules, CA), inside an anaerobic chamber. The voltage was 1.5 kV, and the time constant was 5 ms. The electroporated cells were transferred to 10 ml of complex modified VM medium, and recovered for 24 h at 34°C. The cells were collected by centrifugation, and the cell pellet was spread on complex modified VM agar plates supplemented with 15 μg/ml erythromycin. The plates were incubated at 34°C anaerobically in BD GasPak plastic bags (BD, Franklin Lakes, NJ) for 5 to 7 days until single colonies appeared. To create the *ldh mdh *mutant, the *Ccel*_*2485 *(*ldh*) mutant was transformed using pLyc1217Er0137, and a strain containing an intron insertion in *Ccel_0137 *was isolated.

### Plasmid curing

The donor plasmid pLyc1217Er in mutants was cured by culturing the cells in modified VM liquid medium without erythromycin for 4 to 5 days with daily subculture. Cells from the final subculture were spread on modified VM agar plates without erythromycin and incubated anaerobically in BD GasPak bags at 34°C. Isolated colonies were screened by colony PCR using plasmid-specific primers pWH199F2 and pintronR1. Colonies lacking PCR products were picked for further verification. The donor plasmids could not be cured from *ack *or *pta *mutants using these methods.

### Southern blotting

*C. cellulolyticum *genomic DNA was extracted using a Wizard Genomic DNA Purification Kit (Promega, Madison, WI). A total of 10 μg of genomic DNA was digested with *Eco*RI, which does not cut the inserted intron fragment, and was separated by agarose gel electrophoresis. The DNA was transferred to Hybond-N nucleic acid transfer membrane (GE Healthcare, Piscataway, NJ) and autocrosslinked using a Stratagene UV Stratalinker 1800 (Agilent, Wilmington, DE), followed by baking at 80°C for 30 minutes. The probe was amplified by PCR using dNTPs mixed with Biotin-11-dUTP (Thermo Scientific Fermentas, Glen Burnie, MD) and intron-specific primers pintronF1 and pintronR1. Hybridization and detection were performed using a North2South Chemiluminescent Hybridization and Detection kit (Thermo Scientific, Rockford, IL), following the manufacturer's instructions.

### Metabolic product analysis

Wild-type and mutant strains were grown in 10 ml of modified VM cellobiose medium or 50 ml of MTC medium. Carbon substrates in these defined media were 5.0 g/l D-glucose, 5.0 g/l D-cellobiose, 5.0 or 10 g/l Avicel PH-105 crystalline cellulose (FMC BioPolymer, Philadelphia, PA), 5.0 g/l D-xylose, 5.0 g/l xylan from birch wood or 10 g/l switchgrass (dry mass; pretreated with diluted sulfuric acid). For fermentation product analyses cultures were sampled after incubation for 5 to 14 days, when fermentation was complete. The samples were filtered through 0.2 μm filters, acidified and analyzed for primary fermentation products (lactate, acetate and ethanol) using HPLC [42]. These data from four strains were compared using the KaleidaGraph program (v. 4.1.2, Synergy Software, Reading, PA) to perform one-way analysis of variance (ANOVA) with Dunnett's multiple comparison test (0.05 significance level).

The extent of substrate conversion to primary fermentation products was calculated from the molar carbon atom ratio of primary fermentation products to substrates, assuming a stoichiometric ratio of 2 lactate, 2 acetate + 2 CO_2 _or 2 ethanol + 2 CO_2 _per glucose equivalent. For switchgrass analysis, conversion efficiency and sugar composition was determined using quantitative saccharification [43].

More detailed metabolic profiles were obtained by GC-MS. Supernatant and cell pellet samples for metabolomic analysis were collected from duplicate stationary phase cultures grown in defined VM medium with 5.0 g/l cellobiose. Aliquots containing 250 μL of supernatant or cell lysate and 10 μL of sorbitol (0.1% w/v) were transferred by pipette to a vial and stored at -20°C overnight. The samples were thawed and concentrated to dryness under a stream of N_2_. The internal sorbitol standard was added to correct for subsequent differences in derivatization efficiency and changes in sample volume during heating. Trimethylsilyl derivatives were prepared from each sample for analysis by GC-MS [42]. The GC-MS data indicated the presence of a number of 2-hydroxyacids. To confirm whether these were induced from 2-oxoacids with reactive carbonyl groups, a test sample was additionally prepared using a double derivatization protocol that preferentially protects carbonyl groups [44]. Briefly, 200 μL of methoxamine reagent was added to the test sample, which was heated at 30°C with stirring for 90 min. Then, 800 μL of *N*-methyl-*N*-(trimethylsilyl)trifluoroacetamide + 1% trichloromethylsilane was added and the sample was heated at 37°C for 30 minutes. The sample was analyzed by GC-MS after 2 h storage at room temperature and again after 1 day.

### Enzymatic activity assays

Wild-type and mutant cells were grown in 100 ml of modified VM cellobiose medium to late log phase (optical density at 600 nm of 0.65 to 0.85). The cells were harvested, washed in buffer containing 50 mM *N*-[Tris(hydroxymethyl)methyl]-2-aminoethanesulfonic acid (TES)-NaOH and 10 mM MgCl_2 _(pH 7.2), and resuspended in 10 ml of buffer. The cells were lysed using a French pressure cell and press (Thermo Electron, Needham Heights, MA) at 10,000 psi. The lysate was centrifuged at 18,000 *g *for 10 min to produce cell-free extract. Total protein measurements were made with the BCA Protein Assay Kit (Thermo Pierce, Rockford, IL), using bovine serum albumin as a standard. Malate dehydrogenase and malic enzyme activities were measured at room temperature using a continuous spectrophotometric assay [45]. Standard malate dehydrogenase reaction mixtures (1 ml) contained buffer (described above), 200 mM KCl, 125 μM NADH, 2 mM sodium oxaloacetate (prepared fresh), and cell-free extract (10 to 14 μg protein). Control reactions without oxaloacetate were used to determine the rate of non-specific NADH oxidation in these samples. Standard malic enzyme reaction mixtures (1 ml) contained buffer, 200 mM KCl, 0.2 mM NADP^+^, 2 mM L-malate, and cell-free extract (14 to 21 μg protein). Control reactions without malate were used to determine the rate of non-specific NADP^+ ^reduction. Lactate dehydrogenase reaction mixtures (1 ml) contained buffer, 200 mM KCl, 1 mM pyruvate, 1 mM fructose-1,6-diphosphate, 0.2 mM NADH, and cell free extract (18 μg protein). Specific activities measured from triplicate technical replicates data from four strains were compared using one-way ANOVA with Dunnett's multiple comparison test (0.05 significance level).

## Competing interests

The authors declare that they have no competing interests.

## Authors' contributions

YL planned and carried out genetic experiments, analyzed all results and jointly drafted the manuscript. TT and NE planned and executed metabolomic experiments and analyzed the results. CH and MR performed metabolic product analyses and analyzed the results. DG performed enzyme activity analyses. JL, CS, AG, YY and DG planned experiments, analyzed results and helped draft the manuscript. All authors reviewed the final manuscript draft.

## Supplementary Material

Additional file 1 **PCR analysis of intron insertions in *Clostridium cellulolyticum mdh *and *ldh *genes**. This file contains an image of an ethidium bromide-stained agarose gel illustrating PCR products from erythromycin-resistant *C. cellulolyticum *colonies. Click here for file

Additional file 2 **Sequences of the *mdh *and *ldh *gene disruptions, and the proposed *ack *and *pta *disruptions**. This file contains diagrams and sequences of the gene disruption constructs created for this project. Click here for file

Additional file 3 **Phylogeny of *Clostridial ldh *and *mdh *paralogs**. This file contains a phylogenetic tree showing the most likely evolutionary history of *mdh *and *ldh *paralogs in *Clostridium cellulolyticum *and related organisms. Click here for file

Additional file 4 **Maximum fermentation product concentrations of *Clostridium cellulolyticum *wild-type and mutant strains grown with different carbon sources**. This file contains a table of primary fermentation product concentrations for strains grown on defined medium with glucose, cellobiose, xylose, xylan, pretreated switchgrass or Avicel. Click here for file

Additional file 5 **Colony PCR screen for intron insertions in the phosphotransacetylase (*pta*) and acetate kinase (*ack*) genes**. This file contains images of ethidium bromide-stained agarose gels illustrating PCR products from erythromycin-resistant *Clostridium cellulolyticum *colonies. Click here for file

Additional file 6 ***Clostridium cellulolyticum *plasmids and strains**. This file contains a list of plasmid vectors and *C. cellulolyticum *strains used in this project, along with a list of relevant features or genotypes. Click here for file

Additional file 7 **Oligonucleotide primers used for targeted mutagenesis**. This file contains a table of all oligonucleotides used in this project. Click here for file
