# Supplementary material for: Combined inactivation of the Clostridium cellulolyticum lactate and malate dehydrogenase genes substantially increases ethanol yield from cellulose and switchgrass fermentations
Source: Biotechnol Biofuels. 2012 Jan 4;5:2. doi: 10.1186/1754-6834-5-2 (PMC3268733; doi:10.1186/1754-6834-5-2)
Supplement: Additional file 1 — PCR analysis of intron insertions in Clostridium cellulolyticum mdh and ldh genes. This file contains an image of an ethidium bromide-stained agarose gel illustrating PCR products from erythromycin-resistant C. cellulolyticum colonies. [file 1754-6834-5-2-S1.PDF]

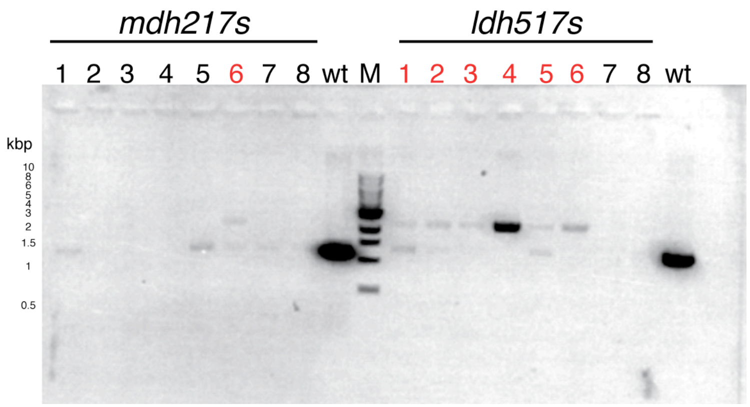

Screening of original erythromycin resistant *C. cellulolyticum* colonies for intron insertions by PCR using gene-specific forward and reverse primers. The numbers at the top are bacterial colony numbers. Red numbers indicate intron insertions. Wild-type (wt) cells were used as controls. The molecular marker (M) lanes contain 1 kb DNA ladder (New England Biolabs): lengths of these standards are indicated on the left. The ethidium bromide-stained gel image was inverted, and brightness, contrast and gamma values were adjusted using Adobe Photoshop CS4.
