# Supplementary material for: Combined inactivation of the Clostridium cellulolyticum lactate and malate dehydrogenase genes substantially increases ethanol yield from cellulose and switchgrass fermentations
Source: Biotechnol Biofuels. 2012 Jan 4;5:2. doi: 10.1186/1754-6834-5-2 (PMC3268733; doi:10.1186/1754-6834-5-2)
Supplement: Additional file 2 — Sequences of the mdh and ldh gene disruptions, and the proposed ack and pta disruptions. This file contains diagrams and sequences of the gene disruption constructs created for this project. [file 1754-6834-5-2-S2.PDF]

## Sequence of the ***mdh*** **(*****Ccel_0137*****) gene disruption by intron**

**A**

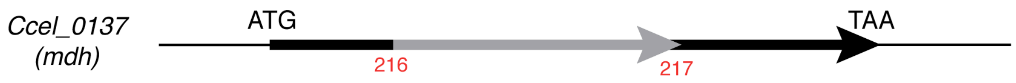


**B**

```
  1 ATGTTAAGGA TTGTGATGTT GTGCGCCCAG ATAGGGTGTT AAGTCAAGTA GTTTAAGGTA
 61 CTACTCTGTA AGATAACACA GAAAACAGCC AACCTAACCG AAAAGCGAAA GCTGATACGG
121 GAACAGAGCA CGGTTGGAAA GCGATGAGTT ACCTAAAGAC AATCGGGTAC GACTGAGTCG
181 CAATGTTAAT CAGATATAAG GTATAAGTTG TGTTTACTGA ACGCAAGTTT CTAATTTCGA
241 TTCAATCTCG ATAGAGGAAA GTGTCTGAAA CCTCTAGTAC AAAGAAAGGT AAGTTAATAA
301 CATCGACTTA TCTGTTATCA CCACATTTGT ACAATCTGTA GGAGAACCTA TGGGAACGAA
361 ACGAAAGCGA TGCCGAGAAT CTGAATTTAC CAAGACTTAA CACTAACTGG GGATACCCTA
421 AACAAGAATG CCTAATAGAA AGGAGGAAAA AGGCTATAGC ACTAGAGCTT GAAAATCTTG
481 CAAGGGTACG GAGTACTCGT AGTAGTCTGA GAAGGGTAAC GCCCTTTACA TGGCAAAGGG
541 GTACAGTTAT TGTGTACTAA AATTAAAAAT TGATTAGGGA GGAAAACCTC AAAATGAAAC
601 CAACAATGGC AATTTTAGAA AGAATCAGTA AAAATTCACA AGAAAATATA GACGAAGTTT
661 TTACAAGACT TTATCGTTAT CTTTTACGTC CAGATATTTA TTACGTGGCG ACGCGTTGGG
721 AAATGGCAAT GATAGCGAAA CAACGTAAAA CTCTTGTTGT ATGCTTTCAT TGTCATCGTC
781 ACGTGATTCA TAAACACAAG TGAATGTCGA CAGTGAATTT TTACGAACGA ACAATAACAG
841 AGCCGTATAC TCCGAGAGGG GTACGTACGG TTCCCGAAGA GGGTGGTGCA AACCAGTCAC
901 AGTAATGTGA ACAAGGCGGT ACCTCCCTAC TTCACATAGT TGTAACTGCA GGGGC
```

A. Diagram of the *Ccel_0137* gene disruption construct.

B. The intron sequence is indicated by arrows. 20 bases of *mdh* gene sequence on each side flanking the intron are boxed, bases 216 and 217 are shown in red, and base modifications of IBS, EBS2 and EBS1d regions specific for *mdh* 216/217 sense insertion are underlined.

**Sequence of the *ldh* (*Ccel_2485*) gene disruption by intron**

**A**

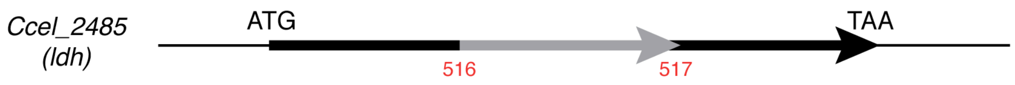


**B**

```
  1 ACCCAAGAAA CGTTCATGCT GTGCGCCCAG ATAGGGTGTT AAGTCAAGTA GTTTAAGGTA
 61 CTACTCTGTA AGATAACACA GAAAACAGCC AACCTAACCG AAAAGCGAAA GCTGATACGG
121 GAACAGAGCA CGGTTGGAAA GCGATGAGTT ACCTAAAGAC AATCGGGTAC GACTGAGTCG
181 CAATGTTAAT CAGATATAAG GTATAAGTTG TGTTTACTGA ACGCAAGTTT CTAATTTCGG
241 TTACGTTCCG ATAGAGGAAA GTGTCTGAAA CCTCTAGTAC AAAGAAAGGT AAGTTATAAG
301 CATGGACTTA TCTGTTATCA CCACATTTGT ACAATCTGTA GGAGAACCTA TGGGAACGAA
361 ACGAAAGCGA TGCCGAGAAT CTGAATTTAC CAAGACTTAA CACTAACTGG GGATACCCTA
421 AACAAGAATG CCTAATAGAA AGGAGGAAAA AGGCTATAGC ACTAGAGCTT GAAAATCTTG
481 CAAGGGTACG GAGTACTCGT AGTAGTCTGA GAAGGGTAAC GCCCTTTACA TGGCAAAGGG
541 GTACAGTTAT TGTGTACTAA AATTAAAAAT TGATTAGGGA GGAAAACCTC AAAATGAAAC
601 CAACAATGGC AATTTTAGAA AGAATCAGTA AAAATTCACA AGAAAATATA GACGAAGTTT
661 TTACAAGACT TTATCGTTAT CTTTTACGTC CAGATATTTA TTACGTGGCG ACGCGTTGGG
721 AAATGGCAAT GATAGCGAAA CAACGTAAAA CTCTTGTTGT ATGCTTTCAT TGTCATCGTC
781 ACGTGATTCA TAAACACAAG TGAATGTCGA CAGTGAATTT TTACGAACGA ACAATAACAG
841 AGCCGTATAC TCCGAGAGGG GTACGTACGG TTCCCGAAGA GGGTGGTGCA AACCAGTCAC
901 AGTAATGTGA ACAAGGCGGT ACCTCCCTAC TTCACTATAT AATCGGTGAA CATGG
```

A. Diagram of the *Ccel_2485* gene disruption construct.

B. The intron sequence is indicated by arrows. 20 bases of *ldh* gene on each side flanking the intron are boxed, bases 516 and 517 are shown in red, and base modifications of IBS, EBS2 and EBS1d regions specific for *ldh* 516/517 sense insertion are underlined.

**Proposed sequence of the *pta369* gene disruption by intron**

**A**

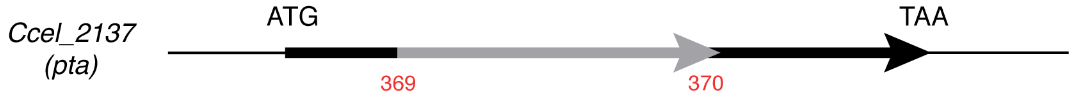


**B**

```
  1 CTGACGGAAT GGTTGCAGGA GTGCGCCCAG ATAGGGTGTT AAGTCAAGTA GTTTAAGGTA
 61 CTACTCTGTA AGATAACACA GAAAACAGCC AACCTAACCG AAAAGCGAAA GCTGATACGG
121 GAACAGAGCA CGGTTGGAAA GCGATGAGTT ACCTAAAGAC AATCGGGTAC GACTGAGTCG
181 CAATGTTAAT CAGATATAAG GTATAAGTTG TGTTTACTGA ACGCAAGTTT CTAATTTCGG
241 TTACCATCCG ATAGAGGAAA GTGTCTGAAA CCTCTAGTAC AAAGAAAGGT AAGTTAGCTC
301 CTGCGACTTA TCTGTTATCA CCACATTTGT ACAATCTGTA GGAGAACCTA TGGGAACGAA
361 ACGAAAGCGA TGCCGAGAAT CTGAATTTAC CAAGACTTAA CACTAACTGG GGATACCCTA
421 AACAAGAATG CCTAATAGAA AGGAGGAAAA AGGCTATAGC ACTAGAGCTT GAAAATCTTG
481 CAAGGGTACG GAGTACTCGT AGTAGTCTGA GAAGGGTAAC GCCCTTTACA TGGCAAAGGG
541 GTACAGTTAT TGTGTACTAA AATTAAAAAT TGATTAGGGA GGAAAACCTC AAAATGAAAC
601 CAACAATGGC AATTTTAGAA AGAATCAGTA AAAATTCACA AGAAAATATA GACGAAGTTT
661 TTACAAGACT TTATCGTTAT CTTTTACGTC CAGATATTTA TTACGTGGCG ACGCGTTGGG
721 AAATGGCAAT GATAGCGAAA CAACGTAAAA CTCTTGTTGT ATGCTTTCAT TGTCATCGTC
781 ACGTGATTCA TAAACACAAG TGAATGTCGA CAGTGAATTT TTACGAACGA ACAATAACAG
841 AGCCGTATAC TCCGAGAGGG GTACGTACGG TTCCCGAAGA GGGTGGTGCA AACCAGTCAC
901 AGTAATGTGA ACAAGGCGGT ACCTCCCTAC TTCACGCAAT TAATTCAACT GCAAA
```

**Intron insertion between bases 369 and 370 of *pta* in the sense orientation, constructed using pLyc1217Er2137-370s.**

A. Diagram of the *Ccel_2137* gene disruption construct.

B. The intron sequence is indicated by arrows. 20 bases of *pta* gene on each side flanking the intron are boxed, bases 369 and 370 are shown in red, and base modifications of EBS2 and EBS1d regions specific for the *pta* 369/370 sense insertion are underlined.

## Proposed sequence of the *pta425* gene disruption by intron

**A**

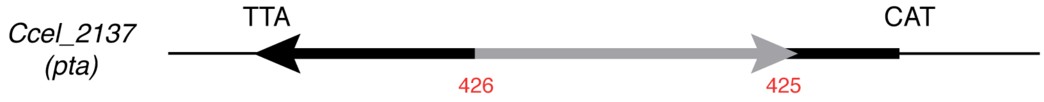


**B**

```
  1 ATACAAGTTT TGCTCCGGGA GTGCGCCCAG ATAGGGTGTT AAGTCAAGTA GTTTAAGGTA
 61 CTACTCTGTA AGATAACACA GAAAACAGCC AACCTAACCG AAAAGCGAAA GCTGATACGG
121 GAACAGAGCA CGGTTGGAAA GCGATGAGTT ACCTAAAGAC AATCGGGTAC GACTGAGTCG
181 CAATGTTAAT CAGATATAAG GTATAAGTTG TGTTTACTGA ACGCAAGTTT CTAATTTCGG
241 TTGCAAATCG ATAGAGGAAA GTGTCTGAAA CCTCTAGTAC AAAGAAAGGT AAGTTAGCTC
301 CCGGGACTTA TCTGTTATCA CCACATTTGT ACAATCTGTA GGAGAACCTA TGGGAACGAA
361 ACGAAAGCGA TGCCGAGAAT CTGAATTTAC CAAGACTTAA CACTAACTGG GGATACCCTA
421 AACAAGAATG CCTAATAGAA AGGAGGAAAA AGGCTATAGC ACTAGAGCTT GAAAATCTTG
481 CAAGGGTACG GAGTACTCGT AGTAGTCTGA GAAGGGTAAC GCCCTTTACA TGGCAAAGGG
541 GTACAGTTAT TGTGTACTAA AATTAAAAAT TGATTAGGGA GGAAAACCTC AAAATGAAAC
601 CAACAATGGC AATTTTAGAA AGAATCAGTA AAAATTCACA AGAAAATATA GACGAAGTTT
661 TTACAAGACT TTATCGTTAT CTTTTACGTC CAGATATTTA TTACGTGGCG ACGCGTTGGG
721 AAATGGCAAT GATAGCGAAA CAACGTAAAA CTCTTGTTGT ATGCTTTCAT TGTCATCGTC
781 ACGTGATTCA TAAACACAAG TGAATGTCGA CAGTGAATTT TTACGAACGA ACAATAACAG
841 AGCCGTATAC TCCGAGAGGG GTACGTACGG TTCCCGAAGA GGGTGGTGCA AACCAGTCAC
901 AGTAATGTGA ACAAGGCGGT ACCTCCCTAC TTCACGCTGT CTTTAATATT TGAAG
```

**Intron insertion between bases 425 and 426 of *pta* in the antisense orientation, constructed using pLyc1217Er2137-426a.**

A. Diagram of the *Ccel_2137* gene disruption construct.

B. The intron sequence is indicated by arrows. 20 bases of *pta* gene on each side flanking the intron are boxed, bases 425 and 426 are shown in red, and base modifications of EBS2 and EBS1d regions specific for the *pta* 425/426 antisense insertion are underlined.

## Proposed sequence of the *pta694* gene disruption by intron

**A**

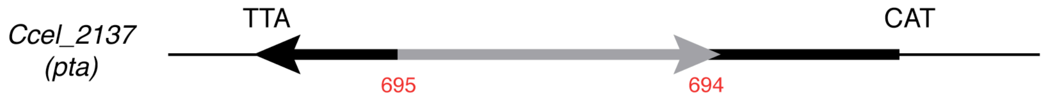


**B**

```
  1 CTGAAGATCT GGAGCTTTTT GTGCGCCCAG ATAGGGTGTT AAGTCAAGTA GTTTAAGGTA
 61 CTACTCTGTA AGATAACACA GAAAACAGCC AACCTAACCG AAAAGCGAAA GCTGATACGG
121 GAACAGAGCA CGGTTGGAAA GCGATGAGTT ACCTAAAGAC AATCGGGTAC GACTGAGTCG
181 CAATGTTAAT CAGATATAAG GTATAAGTTG TGTTTACTGA ACGCAAGTTT CTAATTTCGG
241 TTTCCAGTCG ATAGAGGAAA GTGTCTGAAA CCTCTAGTAC AAAGAAAGGT AAGTTAGGAA
301 AAAGGACTTA TCTGTTATCA CCACATTTGT ACAATCTGTA GGAGAACCTA TGGGAACGAA
361 ACGAAAGCGA TGCCGAGAAT CTGAATTTAC CAAGACTTAA CACTAACTGG GGATACCCTA
421 AACAAGAATG CCTAATAGAA AGGAGGAAAA AGGCTATAGC ACTAGAGCTT GAAAATCTTG
481 CAAGGGTACG GAGTACTCGT AGTAGTCTGA GAAGGGTAAC GCCCTTTACA TGGCAAAGGG
541 GTACAGTTAT TGTGTACTAA AATTAAAAAT TGATTAGGGA GGAAAACCTC AAAATGAAAC
601 CAACAATGGC AATTTTAGAA AGAATCAGTA AAAATTCACA AGAAAATATA GACGAAGTTT
661 TTACAAGACT TTATCGTTAT CTTTTACGTC CAGATATTTA TTACGTGGCG ACGCGTTGGG
721 AAATGGCAAT GATAGCGAAA CAACGTAAAA CTCTTGTTGT ATGCTTTCAT TGTCATCGTC
781 ACGTGATTCA TAAACACAAG TGAATGTCGA CAGTGAATTT TTACGAACGA ACAATAACAG
841 AGCCGTATAC TCCGAGAGGG GTACGTACGG TTCCCGAAGA GGGTGGTGCA AACCAGTCAC
901 AGTAATGTGA ACAAGGCGGT ACCTCCCTAC TTCACCCTTT GCCCCGGGTG TGGCC
```

**Intron insertion between bases 694 and 695 of *pta* in the antisense orientation, constructed using pLyc1217Er2137-695a.**

A. Diagram of the *Ccel_2137* gene disruption construct.

B. The intron sequence is indicated by arrows. 20 bases of *pta* gene on each side flanking the intron are boxed, bases 694 and 695 are shown in red, and base modifications of EBS2 and EBS1d regions specific for the *pta* 694/695 antisense insertion are underlined.

## Proposed sequence of the *pta700* gene disruption by intron

**A**

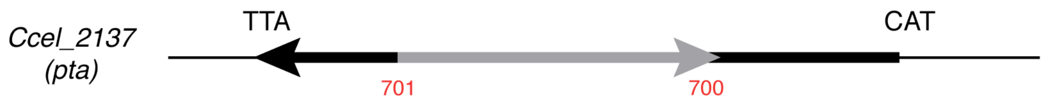


**B**

```
  1 ATCTAACTGA AGATCTGGAG GTGCGCCCAG ATAGGGTGTT AAGTCAAGTA GTTTAAGGTA
 61 CTACTCTGTA AGATAACACA GAAAACAGCC AACCTAACCG AAAAGCGAAA GCTGATACGG
121 GAACAGAGCA CGGTTGGAAA GCGATGAGTT ACCTAAAGAC AATCGGGTAC GACTGAGTCG
181 CAATGTTAAT CAGATATAAG GTATAAGTTG TGTTTACTGA ACGCAAGTTT CTAATTTCGA
241 TTTCTTCTCG ATAGAGGAAA GTGTCTGAAA CCTCTAGTAC AAAGAAAGGT AAGTTAAGCT
301 CCAGGACTTA TCTGTTATCA CCACATTTGT ACAATCTGTA GGAGAACCTA TGGGAACGAA
361 ACGAAAGCGA TGCCGAGAAT CTGAATTTAC CAAGACTTAA CACTAACTGG GGATACCCTA
421 AACAAGAATG CCTAATAGAA AGGAGGAAAA AGGCTATAGC ACTAGAGCTT GAAAATCTTG
481 CAAGGGTACG GAGTACTCGT AGTAGTCTGA GAAGGGTAAC GCCCTTTACA TGGCAAAGGG
541 GTACAGTTAT TGTGTACTAA AATTAAAAAT TGATTAGGGA GGAAAACCTC AAAATGAAAC
601 CAACAATGGC AATTTTAGAA AGAATCAGTA AAAATTCACA AGAAAATATA GACGAAGTTT
661 TTACAAGACT TTATCGTTAT CTTTTACGTC CAGATATTTA TTACGTGGCG ACGCGTTGGG
721 AAATGGCAAT GATAGCGAAA CAACGTAAAA CTCTTGTTGT ATGCTTTCAT TGTCATCGTC
781 ACGTGATTCA TAAACACAAG TGAATGTCGA CAGTGAATTT TTACGAACGA ACAATAACAG
841 AGCCGTATAC TCCGAGAGGG GTACGTACGG TTCCCGAAGA GGGTGGTGCA AACCAGTCAC
901 AGTAATGTGA ACAAGGCGGT ACCTCCCTAC TTCACCTTTT TCCTTTGCCC CGGGT
```

**Intron insertion between bases 700 and 701 of *pta* in the antisense orientation, constructed using pLyc1217Er2137-701a.**

A. Diagram of the *Ccel_2137* gene disruption construct.

B. The intron sequence is indicated by arrows. 20 bases of *pta* gene on each side flanking the intron are boxed, bases 700 and 701 are shown in red, and base modifications of EBS2 and EBS1d regions specific for the *pta* 700/701 antisense insertion are underlined.

**Proposed sequence of the *ack435* gene disruption by intron**

**A**

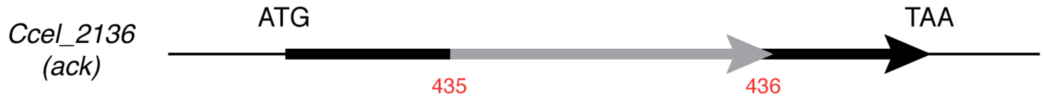


**B**

```
  1 CCAATATACC TATGGTAGCT GTGCGCCCAG ATAGGGTGTT AAGTCAAGTA GTTTAAGGTA
 61 CTACTCTGTA AGATAACACA GAAAACAGCC AACCTAACCG AAAAGCGAAA GCTGATACGG
121 GAACAGAGCA CGGTTGGAAA GCGATGAGTT ACCTAAAGAC AATCGGGTAC GACTGAGTCG
181 CAATGTTAAT CAGATATAAG GTATAAGTTG TGTTTACTGA ACGCAAGTTT CTAATTTCGG
241 TTATAGGTCG ATAGAGGAAA GTGTCTGAAA CCTCTAGTAC AAAGAAAGGT AAGTTAACAG
301 CTACGACTTA TCTGTTATCA CCACATTTGT ACAATCTGTA GGAGAACCTA TGGGAACGAA
361 ACGAAAGCGA TGCCGAGAAT CTGAATTTAC CAAGACTTAA CACTAACTGG GGATACCCTA
421 AACAAGAATG CCTAATAGAA AGGAGGAAAA AGGCTATAGC ACTAGAGCTT GAAAATCTTG
481 CAAGGGTACG GAGTACTCGT AGTAGTCTGA GAAGGGTAAC GCCCTTTACA TGGCAAAGGG
541 GTACAGTTAT TGTGTACTAA AATTAAAAAT TGATTAGGGA GGAAAACCTC AAAATGAAAC
601 CAACAATGGC AATTTTAGAA AGAATCAGTA AAAATTCACA AGAAAATATA GACGAAGTTT
661 TTACAAGACT TTATCGTTAT CTTTTACGTC CAGATATTTA TTACGTGGCG ACGCGTTGGG
721 AAATGGCAAT GATAGCGAAA CAACGTAAAA CTCTTGTTGT ATGCTTTCAT TGTCATCGTC
781 ACGTGATTCA TAAACACAAG TGAATGTCGA CAGTGAATTT TTACGAACGA ACAATAACAG
841 AGCCGTATAC TCCGAGAGGG GTACGTACGG TTCCCGAAGA GGGTGGTGCA AACCAGTCAC
901 AGTAATGTGA ACAAGGCGGT ACCTCCCTAC TTCACGTATT TGATACCACT TTCCA
```

**Intron insertion between bases 435 and 436 of *ack* in the sense orientation, constructed using pLyc1217Er2136-436s.**

A. Diagram of the *Ccel_2136* gene disruption construct.

B. The intron sequence is indicated by arrows. 20 bases of *ack* gene on each side flanking the intron are boxed, bases 435 and 436 are shown in red, and base modifications of EBS2 and EBS1d regions specific for the *pta* 435/436 sense insertion are underlined.
