# Supplementary material for: Combined inactivation of the Clostridium cellulolyticum lactate and malate dehydrogenase genes substantially increases ethanol yield from cellulose and switchgrass fermentations
Source: Biotechnol Biofuels. 2012 Jan 4;5:2. doi: 10.1186/1754-6834-5-2 (PMC3268733; doi:10.1186/1754-6834-5-2)
Supplement: Additional file 3 — Phylogeny of Clostridial ldh and mdh paralogs. This file contains a phylogenetic tree showing the most likely evolutionary history of mdh and ldh paralogs in Clostridium cellulolyticum and related organisms. [file 1754-6834-5-2-S3.PDF]

Phylogeny of *Clostridial ldh* and *mdh* paralogs

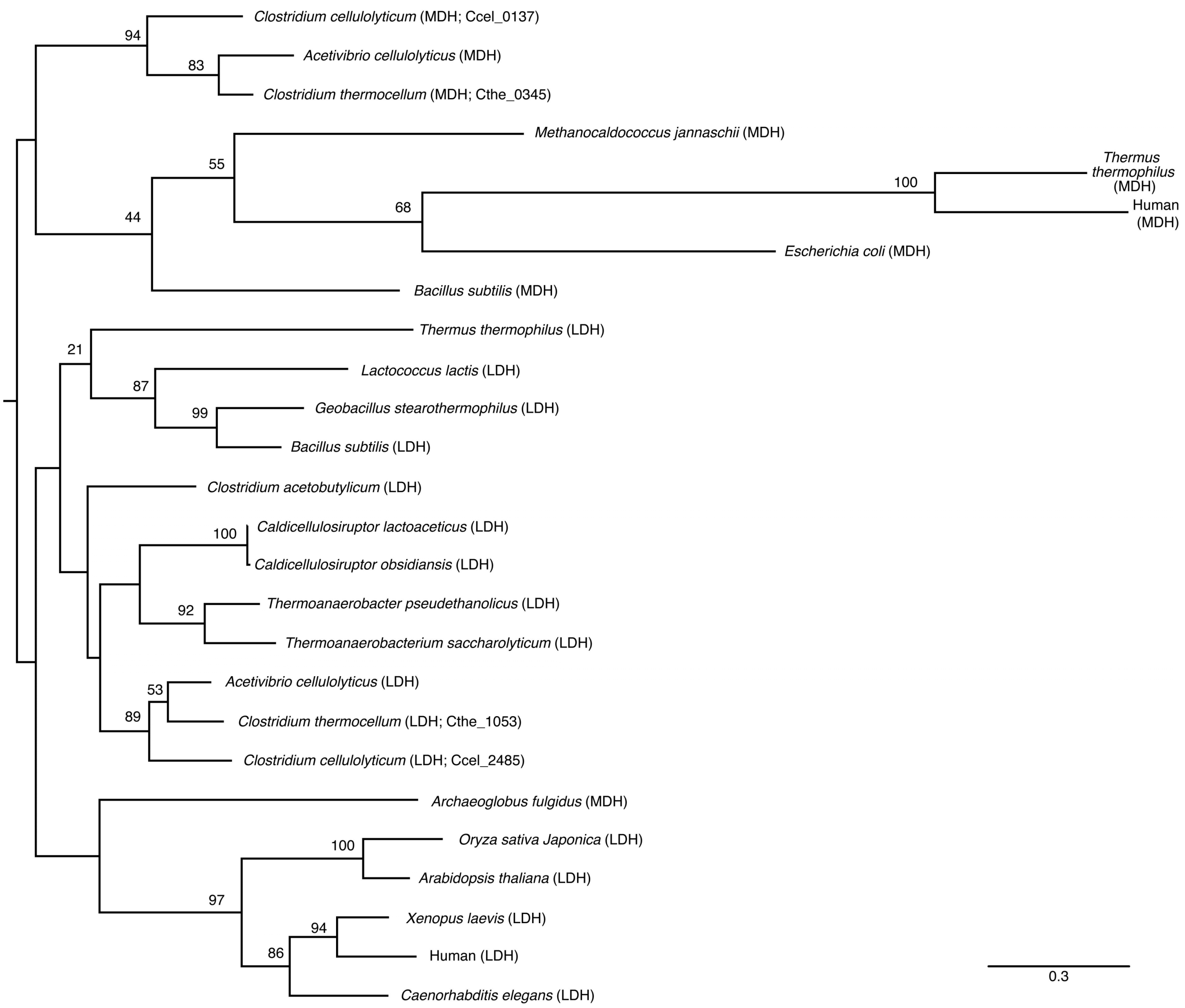

The *C. thermocellum* MDH and LDH paralogs share 46% amino acid identity. The most likely phylogeny of MDH and LDH protein sequences indicates that the clostridial homologs evolved separately, outside the clostridial lineage. An alignment of 26 protein sequences was constructed using the T-Coffee program (ver. 8.99) with default settings. Those sequences and their GenBank, EMBL or PDB accession numbers were *Acetivibrio cellulolyticus* MDH (gi|303242245), *Acetivibrio cellulolyticus* LDH (gi|303239939), *Arabidopsis thaliana* (gi|15236012), *Archaeoglobus fulgidus* MDH (pdb|2X0I), *Bacillus subtilis* LDH (gi|238054375), *Bacillus subtilis* MDH (UNIRef100_P49814), *Caenorhabditis elegans* (gi|17535107), *Caldicellulosiruptor lactoaceticus* (gi|312876463), *Caldicellulosiruptor obsidiansis* (tr|D9TFN5), *Clostridium acetobutylicum* (sp|Q97MD1), *C. cellulolyticum* LDH (*Ccel_2485*; gi|220929884), *C. cellulolyticum* MDH (*Ccel_0137*; gi|220927596), *Clostridium thermocellum* LDH (Cthe_1053; gi|125973568), *Clostridium thermocellum* MDH (Cthe_0345; gi|125972866), *Escherichia coli* MDH (pdb|3HHP), *Geobacillus stearothermophilus* LDH (sp|P00344), *Homo sapiens* LDH (sp|P07864), *Homo sapiens* MDH (sp|P40925), *Lactococcus lactis* LDH (sp|Q01462.3), *Methanocaldococcus jannaschii* MDH (pdb|1HYG), *Oryza sativa* Japonica

(gi|115443679), *Thermoanaerobacter pseudethanolicus* (gi|167038381), *Thermoanaerobacter saccharolyticum* LDH (gi|49035977), *Thermus thermophilis* LDH (pdb| 2V6M), *Thermus thermophilis* MDH (sp|P61977), and *Xenopus laevis* (gi|147898618). This alignment was manually masked using the Jalview program (ver. 1.0) to remove positions with low alignment confidence, producing an alignment with 282 amino acid positions. The proml program from the Phylip package (ver. 3.69) was used to infer the phylogeny, with the JTT model of amino acid replacements and three HMM states (α=2.8). The seqboot program (Phylip package) was used to prepare 500 bootstrap replicate alignments, and the proml program was again used to infer 500 phylogenies. The consense program (Phylip) constructed a consensus tree showing the plurality of replicates supporting each branch on this phylogram. The scale bar indicates 0.3 amino acid replacements per position. Protein function predictions were assigned manually based on conserved amino acid residues or experimental evidence listed in sequence databases.
