# Supplementary material for: Combined inactivation of the Clostridium cellulolyticum lactate and malate dehydrogenase genes substantially increases ethanol yield from cellulose and switchgrass fermentations
Source: Biotechnol Biofuels. 2012 Jan 4;5:2. doi: 10.1186/1754-6834-5-2 (PMC3268733; doi:10.1186/1754-6834-5-2)
Supplement: Additional file 4 — Maximum fermentation product concentrations of Clostridium cellulolyticum wild-type and mutant strains grown with different carbon sources. This file contains a table of primary fermentation product concentrations for strains grown on defined medium with glucose, cellobiose, xylose, xylan, pretreated switchgrass or Avicel. [file 1754-6834-5-2-S4.PDF]

Maximum fermentation product concentrations of *C. cellulolyticum* wild-type (WT) and mutant strains grown with different carbon sources

Medium	Strains	Lactate (g/L)	Acetate (g/L)	Ethanol (g/L)
VM glucose (5.0 g/L)	WT	0.185±0.021	0.230±0.004	0.393±0.020
	mdh	0.326±0.018	0.200±0.020	0.375±0.039
	ldh	0.028±0.006	0.296±0.011	0.438±0.058
	ldh mdh	0.013±0.001	0.121±0.006	0.454±0.021
VM cellobiose (5.0 g/L)	WT	0.989±0.221	0.536±0.057	0.544±0.049
	mdh	1.406±0.067	0.441±0.034	0.642±0.042
	ldh	0.091±0.010	0.747±0.041	0.703±0.026
	ldh mdh	0.031±0.002	0.386±0.016	1.178±0.072
VM cellulose (10.0 g/L)	WT	1.662±0.163	1.183±0.050	0.312±0.031
	mdh	1.341±0.121	0.608±0.066	0.522±0.053
	ldh	0.107±0.012	1.121±0.038	0.536±0.072
	ldh mdh	0.029±0.010	0.215±0.032	2.650±0.106
VM xylose (5.0 g/L)	WT	0.831±0.049	0.635±0.036	0.404±0.074
	mdh	0.719±0.049	0.255±0.011	0.377±0.004
	ldh	0.043±0.004	0.671±0.013	0.393±0.042
	ldh mdh	0.030±0.006	0.505±0.018	0.401±0.021
VM xylan (5.0 g/L)	WT	0.173±0.014	1.128±0.153	0.202±0.005
	mdh	0.170±0.017	0.534±0.034	0.186±0.010
	ldh	0.026±0.001	1.107±0.107	0.369±0.017
	ldh mdh	0.017±0.001	0.645±0.057	0.565±0.027
VM pretreated switchgrass (10.0 g/L)	WT	0.220±0.045	1.760±0.031	0.327±0.020
	ldh mdh	0.021±0.001	1.058±0.023	1.299±0.050
MTC cellobiose (5.0 g/L)	WT	0.182±0.002	1.355±0.011	0.229±0.013
	ldh mdh	0.015±0.001	0.701±0.003	1.121±0.013
MTC Avicel (5.0 g/L)	WT	0.083±0.016	1.065±0.044	0.257±0.017
	ldh mdh	0.008	0.765±0.004	1.092±0.038
VM cellobiose (5 g/L)	WT	1.361±0.095	0.599±0.029	0.502±0.008
	pta	1.051±0.132	0.417±0.029	0.715±0.023
	ack	1.491±0.127	0.423±0.027	0.660±0.064
