# Supplementary material for: Combined inactivation of the Clostridium cellulolyticum lactate and malate dehydrogenase genes substantially increases ethanol yield from cellulose and switchgrass fermentations
Source: Biotechnol Biofuels. 2012 Jan 4;5:2. doi: 10.1186/1754-6834-5-2 (PMC3268733; doi:10.1186/1754-6834-5-2)
Supplement: Additional file 5 — Colony PCR screen for intron insertions in the phosphotransacetylase (pta) and acetate kinase (ack) genes. This file contains images of ethidium bromide-stained agarose gels illustrating PCR products from erythromycin-resistant Clostridium cellulolyticum colonies. [file 1754-6834-5-2-S5.PDF]

Colony PCR screen for intron insertions in the phosphotransacetylase (*pta*) gene.

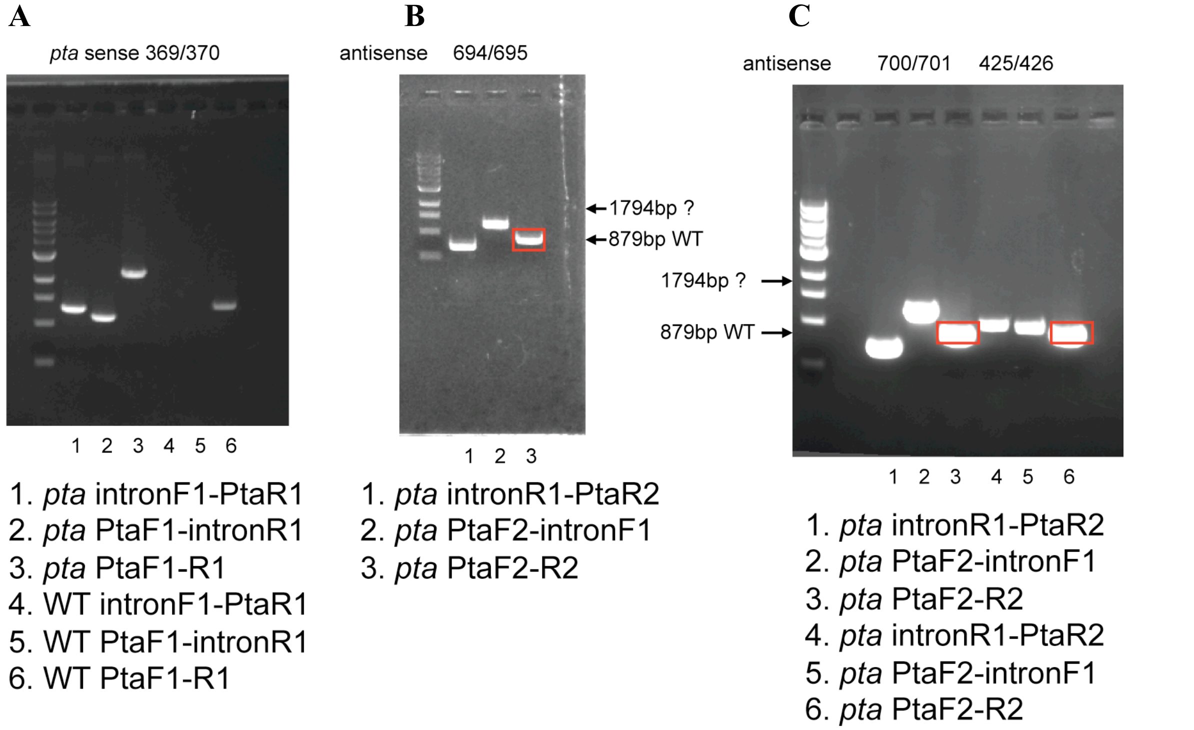

The introns were targeted in the sense orientation (A) or antisense orientation (B and C).

Part A: Confirmation of pure cultures of the *pta* mutants by PCR, using different combination of primers. Amplification of the 5′ and 3′ intron-*pta* junction regions using one primer in the genome and the other in the intron (intronF1/PtaR1 for the 3′ junction and PtaF1/intronR1 for the 5′ junction) resulted in bands from the *pta* mutant (lanes 1 and 2), but not in wild-type cells (lanes 4 and 5). In PCR reactions using PtaF1/PtaR1 primers, the mutant showed a single band (lane 3), which was 915-bp larger than the single band (lane 6) in wild-type cells, confirming the expected intron insertion.

Part B and C: For the antisense insertion, primers PtaF2/intronF1 (5′ junction) and intronR1/PtaR2 (3′ junction) produced bands (B: lanes 1 and 2, C: lane 1, 2, 4 and 5). But using PtaF2/R2, only wild-type bands (879 bp, boxed) were amplified (B: lane 3, C: lane 3 and 6).

Colony PCR screen for intron insertions in the acetate kinase (*ack*) gene.

ak sense 435/436

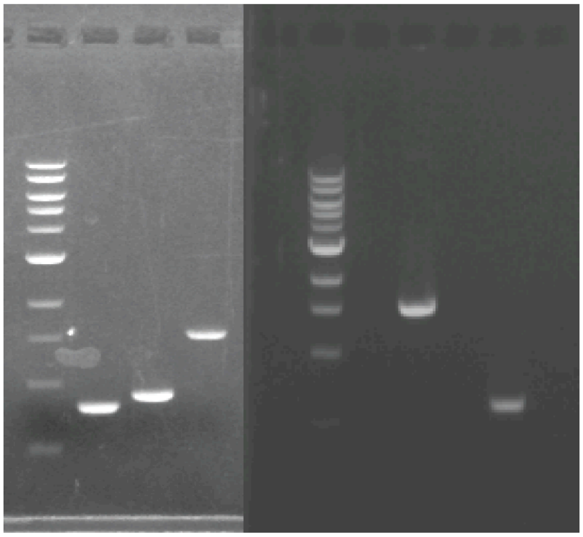

1 2 3 4 5

1. *ak* AkF-intronR1
2. *ak* intronF1-AkR
3. *ak* AkF-R
4. *ak* AkF-R
5. WT AkF-R

The introns were targeted in the sense orientation. With the combination of gene-specific and intron-specific primers, bands were amplified for *ack* mutants (lane 1 and 2). With gene-specific primers, the mutant showed a single band (lane 3 and 4), which was 915-bp larger than the single band (lane 5) in wild-type cells, confirming the expected intron insertion.
