# Supplementary material for: Combined inactivation of the Clostridium cellulolyticum lactate and malate dehydrogenase genes substantially increases ethanol yield from cellulose and switchgrass fermentations
Source: Biotechnol Biofuels. 2012 Jan 4;5:2. doi: 10.1186/1754-6834-5-2 (PMC3268733; doi:10.1186/1754-6834-5-2)
Supplement: Additional file 6 — Clostridium cellulolyticum plasmids and strains. This file contains a list of plasmid vectors and C. cellulolyticum strains used in this project, along with a list of relevant features or genotypes. [file 1754-6834-5-2-S6.PDF]

Clostridium cellulolyticum plasmids and strains

Plasmid	Relevant features	Source
pWH199	Er^R Kan^R pAMβ1 origin, pMB1 origin, Fd promoter	Higashide W *et al*. (2011)
pJIR750ai	L1.LtrB-ΔORF intron, *ltrA*	Chen Y *et al*. (2005)
pLyc1217Er	pWH199 with intron+*ltrA*	This study
pLyc1217Er0137	pLyc1217Er with intron retargeting *Ccel_0137*	This study
pLyc1217Er2137-370s	pLyc1217Er with intron retargeting *Ccel_2137*	This study
pLyc1217Er2137-695a	pLyc1217Er with intron retargeting *Ccel_2137*	This study
pLyc1217Er2137-701a	pLyc1217Er with intron retargeting *Ccel_2137*	This study
pLyc1217Er2137-426a	pLyc1217Er with intron retargeting *Ccel_2137*	This study
pLyc1217Er2136-436s	pLyc1217Er with intron retargeting *Ccel_2136*	This study
pLyc1217Er2485	pLyc1217Er with intron retargeting *Ccel_2485*	This study
**Strain**	**Relevant genotype**	**Source**
C. cellulolyticum H10	Wild-type	ATCC 35319
mdh mutant	*Ccel_0137*::LtrB	This study
ldh mutant	*Ccel_2485*::LtrB	This study
ldh mdh mutant	*Ccel_2485*::LtrB *Ccel_0137*::LtrB	This study
pta mutant	*Ccel_2137*::LtrB (pLyc1217Er2137-370s)	This study
ack mutant	*Ccel_2136*::LtrB (pLyc1217Er2136-436s)	This study

Higashide W, Li Y, Yang Y, Liao JC. 2011. Metabolic engineering of *Clostridium cellulolyticum* for production of isobutanol from cellulose. Appl Environ Microbiol 77(8):2727-2733.

Chen Y, McClane BA, Fisher DJ, Rood JI, Gupta P. 2005. Construction of an alpha toxin gene knockout mutant of *Clostridium perfringens* Type A by use of a mobile group II intron. Appl Environ Microbiol 71(11):7542-7547.
