# Supplementary material for: Combined inactivation of the Clostridium cellulolyticum lactate and malate dehydrogenase genes substantially increases ethanol yield from cellulose and switchgrass fermentations
Source: Biotechnol Biofuels. 2012 Jan 4;5:2. doi: 10.1186/1754-6834-5-2 (PMC3268733; doi:10.1186/1754-6834-5-2)
Supplement: Additional file 7 — Oligonucleotide primers used for targeted mutagenesis. This file contains a table of all oligonucleotides used in this project. [file 1754-6834-5-2-S7.PDF]

Oligonucleotide primers used for targeted mutagenesis

Primer name	Sequence[1]	Function
pJIR750aiXmaIF	CAAAACCCGGGATAATTATCCTTACCAGCCCATAGGGTGCGCCCAGATAGG[1]	Forward primer to clone intron+ltrA from pJIR750ai
pJIR750aiXhoIR	CAAAACTCGAGCGGGTGCTCGACATTCACTTGTGTTTATGAATCACGTGAC	Reverse primer to clone intron+ltrA from pJIR750ai
EBSu	CGAAATTAGAAACTTGCGTTCAGTAAAC	EBS universal primer for cross-over PCR
Mdh217sIBS	AAAACCCGGGATAATTATCCTTAGATTGCGATGTTGTGCGCCCAGATAGGGTG	IBS primer for cross-over PCR to retarget *Ccel_0137*
Mdh217sEBS1d	CAGATTGTACAAATGTGGTGATAACAGATAAGTCGATGTTATTAACTTACCTTTCTTTGT	EBS1d primer for cross-over PCR to retarget *Ccel_0137*
Mdh217sEBS2	TGAACGCAAGTTTCTAATTTCGATTCAATCTCGATAGAGGAAAGTGTCT	EBS2 primer for cross-over PCR to retarget *Ccel_0137*
Ldh517sIBS	AAAACCCGGGATAATTATCCTTAAACGTCCATGCTGTGCGCCCAGATAGGGTG	IBS primer for cross-over PCR to retarget *Ccel_2485*
Ldh517sEBS1d	CAGATTGTACAAATGTGGTGATAACAGATAAGTCCATGCTTATAACTTACCTTTCTTTGT	EBS1d primer for cross-over PCR to retarget *Ccel_2485*
Ldh517sEBS2	TGAACGCAAGTTTCTAATTTCGGTTACGTTCCGATAGAGGAAAGTGTCT	EBS2 primer for cross-over PCR to retarget *Ccel_2485*
MdhF	GGGATTTTAATGGGTTTTAAAGTTG	Forward primer to screen *Ccel_0137* mutant and sequence
MdhR	TCCAGGTGAATAAGCTAAAGAAAGA	Reverse primer to screen *Ccel_0137* mutant and sequence
LdhF	TATACTTTTGCACCCAGAATGTTTT	Forward primer to screen *Ccel_2485* mutant and sequence
LdhR	TGACTGATACGGGTTTTATCAATTT	Reverse primer to screen *Ccel_2485* mutant and sequence
pWH199F2	ATAAGTTATGGTTGGAATTGTGAGC	pWH199 plasmid specific primer to screen and sequence
pintronF1	CCTATGGGAACGAAACGAAA	Intron-specific primer to screen and sequence
pintronR1	CGAGTACTCCGTACCCTTGC	Intron-specific primer to screen and sequence
Pta370sIBS	AAAACCCGGGATAATTATCCTTAATGGTCGCAGGAGTGCGCCCAGATAGGGTG	IBS primer for cross-over PCR to retarget *Ccel_2137*, sense orientation
Pta370sEBS1d	CAGATTGTACAAATGTGGTGATAACAGATAAGTCGCAGGAGCTAACTTACCTTTCTTTGT	EBS1d primer for cross-over PCR to retarget *Ccel_2137*, sense orientation
Pta370sEBS2	TGAACGCAAGTTTCTAATTTCGGTTACCATCCGATAGAGGAAAGTGTCT	EBS2 primer for cross-over PCR to retarget *Ccel_2137*, sense orientation
Pta695aIBS	AAAACCCGGGATAATTATCCTTACTGGACCTTTTTGTGCGCCCAGATAGGGTG	IBS primer for cross-over PCR to retarget *Ccel_2137*, antisense orientation
Pta695aEBS1d	CAGATTGTACAAATGTGGTGATAACAGATAAGTCCTTTTTCCTAACTTACCTTTCTTTGT	EBS1d primer for cross-over PCR to retarget *Ccel_2137*, antisense orientation
Pta695aEBS2	TGAACGCAAGTTTCTAATTTCGGTTTCCAGTCGATAGAGGAAAGTGTCT	EBS2 primer for cross-over PCR to retarget *Ccel_2137*, antisense orientation
Pta701aIBS	AAAACCCGGGATAATTATCCTTAGAAGACCTGGAGGTGCGCCCAGATAGGGTG	IBS primer for cross-over PCR to retarget *Ccel_2137*, antisense orientation
Pta701aEBS1d	CAGATTGTACAAATGTGGTGATAACAGATAAGTCCTGGAGCTTAACTTACCTTTCTTTGT	EBS1d primer for cross-over PCR to retarget *Ccel_2137*, antisense orientation
Pta701aEBS2	TGAACGCAAGTTTCTAATTTCGATTTCTTCTCGATAGAGGAAAGTGTCT	EBS2 primer for cross-over PCR to retarget *Ccel_2137*, antisense orientation
Pta426aIBS	AAAACCCGGGATAATTATCCTTATTTGCCCCGGGAGTGCGCCCAGATAGGGTG	IBS primer for cross-over PCR to retarget *Ccel_2137*, antisense orientation
Pta426aEBS1d	CAGATTGTACAAATGTGGTGATAACAGATAAGTCCCGGGAGCTAACTTACCTTTCTTTGT	EBS1d primer for cross-over PCR to retarget *Ccel_2137*, antisense orientation
Pta426aEBS2	TGAACGCAAGTTTCTAATTTCGGTTGCAAATCGATAGAGGAAAGTGTCT	EBS2 primer for cross-over PCR to retarget *Ccel_2137*, antisense orientation
Ack436sIBS	AAAACCCGGGATAATTATCCTTACCTATCGTAGCTGTGCGCCCAGATAGGGTG	IBS primer for cross-over PCR to retarget *Ccel_2136*, sense orientation
Ack436sEBS1d	CAGATTGTACAAATGTGGTGATAACAGATAAGTCGTAGCTGTTAACTTACCTTTCTTTGT	EBS1d primer for cross-over PCR to retarget *Ccel_2136*, sense orientation
Ack436sEBS2	TGAACGCAAGTTTCTAATTTCGGTTATAGGTCGATAGAGGAAAGTGTCT	EBS2 primer for cross-over PCR to retarget *Ccel_2136*, sense orientation
PtaF1	TATAACAGTTTCAACGGTTTGTGAA	Forward primer to screen *Ccel_2137* mutant and sequence
PtaR1	AACTCCCCGCATTTATAACTAAAAC	Reverse primer to screen *Ccel_2137* mutant and sequence
PtaF2	CTTCCTGAAAGTAACGATATCAGAA	Forward primer to screen *Ccel_2137* mutant and sequence
PtaR2	TACAGCCTCTTGAAAGGTCATTAAC	Reverse primer to screen *Ccel_2137* mutant and sequence
AckF	GTTTTAGTTATAAATGCGGGGAGTT	Forward primer to screen *Ccel_2136* mutant and sequence
AckR	TGTTAACAGCACAAATACTTGAACC	Reverse primer to screen *Ccel_2136* mutant and sequence

[1] Restriction enzyme sites in primers are underlined.
